# Real‐Time In Vivo Monitoring of Anastomotic Intestinal Ischemia Using Implantable Resorbable Organic Sensors

**DOI:** 10.1002/advs.202514507

**Published:** 2025-12-03

**Authors:** Dennis Wahl, Finn Jaekel, Julia Henne, Richard Kantelberg, Daniel C. Freund, Eberhard Grambow, Brigitte Vollmar, Amelie R. Zitzmann, Hans Kleemann, Jochen Hampe, Sebastian Hinz, Clemens Schafmayer, Karl Leo

**Affiliations:** ^1^ Department of General, Visceral, Thoracic, Vascular and Transplantation Surgery Rostock University Medical Center 18057 Rostock Germany; ^2^ Dresden Integrated Center for Applied Physics and Photonic Materials (IAPP) Technical University of Dresden 01187 Dresden Germany; ^3^ Department of Cardiac, Thoracic and Vascular Surgery University Medical Center Göttingen 37075 Göttingen Germany; ^4^ Rudolf‐Zenker‐Institute for Experimental Surgery University Medical Center Rostock 18057 Rostock Germany; ^5^ Department of Anesthesiology, Intensive Care Medicine and Pain Therapy University Medical Centre of Rostock 18057 Rostock Germany; ^6^ University Hospital Carl Gustav Carus at the Technical University of Dresden 01307 Dresden Germany

**Keywords:** anastomotic leakage, bioimpedance, biosensor, implant, resorbable

## Abstract

Anastomotic failure remains one of the most severe complications in gastrointestinal surgery. Despite continuous advancements in stapler technologies and surgical techniques, it continues to be a leading cause of postoperative morbidity and mortality. It contributes substantially to prolonged hospitalization and increased healthcare expenditures. Currently, diagnosis is based on secondary systemic signs, such as inflammatory response or changes in drain fluid, followed by a multimodal diagnostic approach. However, reliable early detection of local alterations is still lacking. Here, the implantation of a bioresorbable is investigated, intra‐anastomotically placed sensor device. By performing real‐time intra‐anastomotic bioimpedance measurements, ischemia‐related changes are identified at an early, potentially reversible stage, prior to the onset of clinical or systemic manifestations. Furthermore, the sensor device offers the potential for future integration of pattern‐recognition algorithms and the possibility of direct measurement of different markers in the anastomotic microenvironment.

## Graphical abstract

Resorbable impedance sensors are successfully implanted into porcine small intestinal anastomoses. Impedance was recorded for 2 hours prior, and 2 hours following ischemia induction, and a significant drop in tissue impedance was observed.

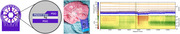

## 1 Introduction

Implantable bioelectronic sensors may enhance the quality of postoperative patient care. This is particularly relevant in the surgical field, where direct monitoring of the surgical site is challenging. Here, novel devices may offer promising complementary solutions by enabling early detection of potentially life‐threatening complications. In gastrointestinal surgery, reconstruction of the intestinal passage following bowel resection, such as in cases of colorectal cancer, is typically achieved through the creation of an anastomosis, performed either by hand‐sewn techniques or with the aid of surgical staplers. Anastomotic leakage (AL), defined as a dehiscence at the anastomotic site, may result in the extravasation of bowel contents into the abdominal cavity. The incidence rate of AL varies depending on the location of the anastomosis. For colorectal surgery, leak rates of 1.5% to 16% are reported, while for esophageal surgery, leak rates range from 8% to 20%. Depending on severity, leaks are associated with significantly increased morbidity and mortality, as well as substantial utilization of hospital resources. Besides short‐term consequences, patients who experienced an AL face a poorer long‐term outcome, including increased local recurrence rates in malignant disease, and a reduced 5‐year survival rate.^[^ ^1^, ^2^, ^3^, ^4^, ^5^, ^6^ ^]^

Despite extensive research, the pathophysiology of AL remains incompletely understood. Known risk factors include patient‐specific and surgical variables such as anastomosis location, surgical technique, and surgeon experience. A key contributor is impaired wound healing, which may result from hypoproteinemia or tissue hypoxia.^[^ ^7^, ^8^, ^9^ ^]^ In particular, inadequate blood supply, due to risk factors like diabetes, smoking, microvascular disease, or excessive tension, has been correlated with increased AL rates.^[^ ^10^, ^11^ ^]^ Insufficient perfusion compromises oxygen delivery, leading to ischemia, tissue necrosis, and ultimately, partial or complete anastomotic dehiscence.^[^ ^12^ ^]^

Until now, the diagnosis of an AL remains a challenge in clinical practice. Current diagnostic approaches rely on radiologic imaging, endoscopy, and the presence of clinical signs. Patients typically present with abdominal pain, changes in drainage fluid, ileus, elevated serum procalcitonin and C‐reactive protein levels, fever, and, in severe cases, peritonitis or sepsis. Importantly, these manifestations often appear several days after the onset of ischemia, delaying the diagnosis to 5–7 days post‐surgery.^[^ ^13^, ^14^ ^]^ Following ischemia, various cellular changes occur, including alterations in pH levels, cell edema, closure of gap junctions, and dysfunction of ion channels. These alterations affect the tissue's electrical properties, which can be quantified via changes in bioimpedance. Bioimpedance monitoring has demonstrated the ability to detect ischemia‐related alterations in tissue conductivity.^[^ ^15^, ^16^, ^17^ ^]^

In this study, we investigated the application of bioimpedance sensor devices integrated onto a resorbable polydioxanone (PDO) substrate for the detection of ischemia, aiming to establish a real‐time early warning system for AL. We previously demonstrated the feasibility and safety of integrating impedance sensors onto PDO substrates.^[^ ^18^ ^]^ Additionally, we confirmed that the incorporation of an additional PDO membrane into the anastomotic site does not impair wound healing, thereby allowing a safe implantation of sensor‐bearing membranes.^[^ ^19^ ^]^ Here, we present in vivo data demonstrating the reliable and stable performance of intra‐anastomotic impedance measurements over several hours at the anastomotic site. Most importantly, we show that an induced ischemia results in a significant and measurable change in tissue impedance. Through a circular sensor configuration, we achieved comprehensive spatial monitoring of ischemia around the entire anastomosis. Moreover, we validated our findings using hyperspectral imaging and observe a trend indicating that the magnitude of impedance change correlates with the severity of ischemia.

## 2 Methods – Sensor Integration and Experimental Design

To translate the concept of bioimpedance‐based ischemia detection into a clinically applicable system, a fully implantable, resorbable sensor platform designed for seamless integration into circular‐stapled intestinal anastomoses was developed. In the following sections, we present the technical realization of this approach, alongside in vivo validation in a porcine model to evaluate its performance under physiological conditions.

### 2.1 Sensor Design

To enable reliable bioimpedance measurements at the anastomotic site, a custom sensor device was developed, focusing on both mechanical integration and electrical performance.

The shape and geometry of the substrate are designed to be mechanically compatible with standard surgical procedures using a 21 mm Ethicon^®^ circular stapler (Norderstedt, Germany) used for creation of the anastomosis. An outer ring, measuring 28.88 mm, is connected by 12 spokes (0.6 mm or 2 mm wide) to a central ring, ensuring reproducible positioning and alignment of the sensor on the stapler. The central ring is removed during stapling, while the spokes anchor the sensor in the intestinal wall. This specific design has been shown to have minimal effect on the anastomotic healing process when implanted into the anastomotic site.^[^ ^19^ ^]^ The substrate material is medical‐grade PDO, a bioresorbable polymer widely used in surgical sutures, chosen for its mechanical stability, compatibility with established sterilization methods, and its predictable resorption kinetics.

The sensing electrodes are arranged in three pairs of two on the 2 mm wide spokes, optimizing the available space and signal integrity. An additional resistive reference structure in the shape of a meandering conductive line is integrated. The design is constrained by the used data acquisition system, which allows for impedance measurements at four channels simultaneously. With the reference resistor occupying one channel, bioimpedance can be measured at three locations per implant.

**Figure 1:**
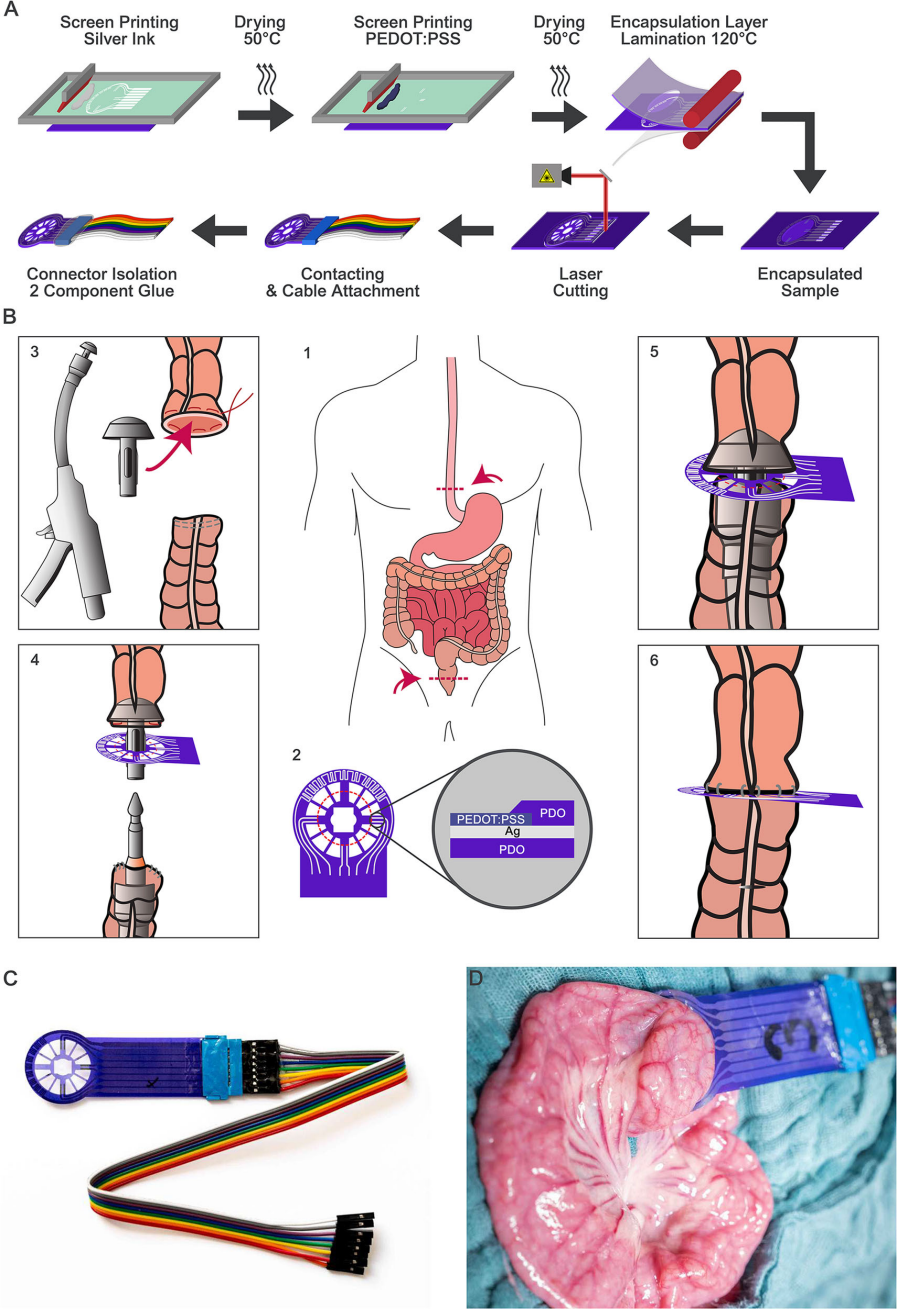
A) Sensor fabrication process. Silver and PEDOT:PSS layers are screen‐printed and dried at 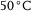. An additional PDO encapsulation layer is laminated onto the substrate. Finished samples are cut using a high‐precision laser structuring machine. Finally, the samples are contacted, and the contact is isolated using 2‐component glue. B) Implantation procedure: *1*: Envisioned implantation sites at the esophagus or colon. *2*: Sketch and cross‐section of the printed anastomosis sensors, red dashed line marks the cutout *3*: The anvil is inserted at the oral end of the resected bowel and is fixed using a purse‐string suture. The shaft of the stapler is inserted through an enterotomy into the aboral end. *4*: The finished sensor is slid between the anvil and the shaft. *5*: Both stapler ends are brought together and the anastomosis is fired. *6*: The stapler is removed, and an intact anastomosis with an implanted sensor is created. C) Optical images of a finished sensor already contacted with an Amphenol Clincher FPC connector and standard 20 cm jumper cables. D) Image of an implanted sensor in the small intestine.

Sensors are fabricated by screen printing silver‐based conductive ink onto PDO sheets, followed by a screen‐printed layer of PEDOT:PSS on the electrodes to enhance electrode‐tissue coupling. A second PDO layer is laminated to encapsulate and protect the conductive structures.^[^ ^18^ ^]^ The final sensor geometry is defined by laser cutting, and external electrical connections are established through FPC connectors. Epoxy glue is used to mechanically seal and electrically isolate the connector region. The process is depicted in **Figure** **1**.

Full technical specifications, including geometrical dimensions, electrode layout, and fabrication steps are provided in the Supporting Information (S2.1.1–S2.2.3)

### 2.2 Experimental Procedure

#### 2.2.1 Ethical Statement

All animal experiments were approved by the German local authority: Landesamt für Landwirtschaft, Lebensmittelsicherheit und Fischerei Mecklenburg‐Vorpommern (approval no. 7221.3‐1‐050/19), under the German animal protection law and the EU Guideline 2010/63/EU. Four German Landrace pigs (age 15 weeks to 18 weeks; 38.0kgto49.6kg) were kept under standardized conditions at the central animal facility of the University Medical Center in Rostock for a seven‐day acclimatization period. During this time, animals were monitored by veterinary staff. 24 hours prior to surgery, animals received water only. Premedication and general anesthesia were performed according to institutional protocols established by the Institute for Experimental Surgery.^[^ ^20^ ^]^ Information on the exact medication is found in the Supporting Information (S2.3.1).

Following the initial evaluation of our sensor model and surgical technique in two animals, we developed a standardized protocol for a reproducible ischemia model (compare detailed explanation in the Supporting Information). A total of seven anastomoses were created across both animals, resulting in data acquisition from 21 sensors.

#### 2.2.2 Surgical Model

After sterile preparation, a midline laparotomy is performed, and side‐to‐end ileoileal anastomoses are created using a circular stapler. The anvil of the stapler is inserted into the proximal end of the bowel and secured with a purse‐string suture (Vicryl 3.0, Ethicon, Norderstedt, Germany), while the shaft is placed through an enterotomy into the distal bowel limb. The sensor is positioned between the anvil and the shaft prior to firing, integrating it directly into the anastomosis (compare Figure 1B.3–1B.6). Through the cutting of the central part of the sensor membrane, an intraluminal mucosal overlap is achieved. After closure of the enterotomy, all sensors are inspected for dislocation, and blood perfusion at the anastomotic site is verified using hyperspectral imaging. Segmental ischemia is induced by tying pre‐positioned ligatures around the supplying mesenteric vessels of the anastomotic segment. Impedance spectra are recorded continuously at minimum 120 min before and after ischemia onset. During this period, the abdomen is covered with moistened swabs to preserve physiological conditions. After ending the impedance measurements, hyperspectral imaging is used again to verify ischemia, using TIVITA Tissue.^[^ ^21^, ^22^ ^]^ Full procedural details are found in the Supporting Information, Section 2.

#### 2.2.3 Impedance Measurements

Bioimpedance is measured using a custom setup based on a high‐speed USB data acquisition (DAQ) device (MCC USB‐1208HS‐4AO) and a custom programming script. A sinusoidal voltage signal with an amplitude of 0.1 V is applied across a 2.2 kΩ reference resistor in series with a sensor electrode pair. Impedance spectra are acquired continuously every 30 s at frequencies between 1 Hz to 25 kHz. One electrode pair of one anastomosis is used for validation of the recorded in vivo data using a high‐precision laboratory‐grade instrument (Autolab PG‐STAT302N, Metrohm AG, Switzerland). For more details, see Supporting Information (S2.3.3).

## 3 Results

### 3.1 Baseline Stability

**Figure 2:**
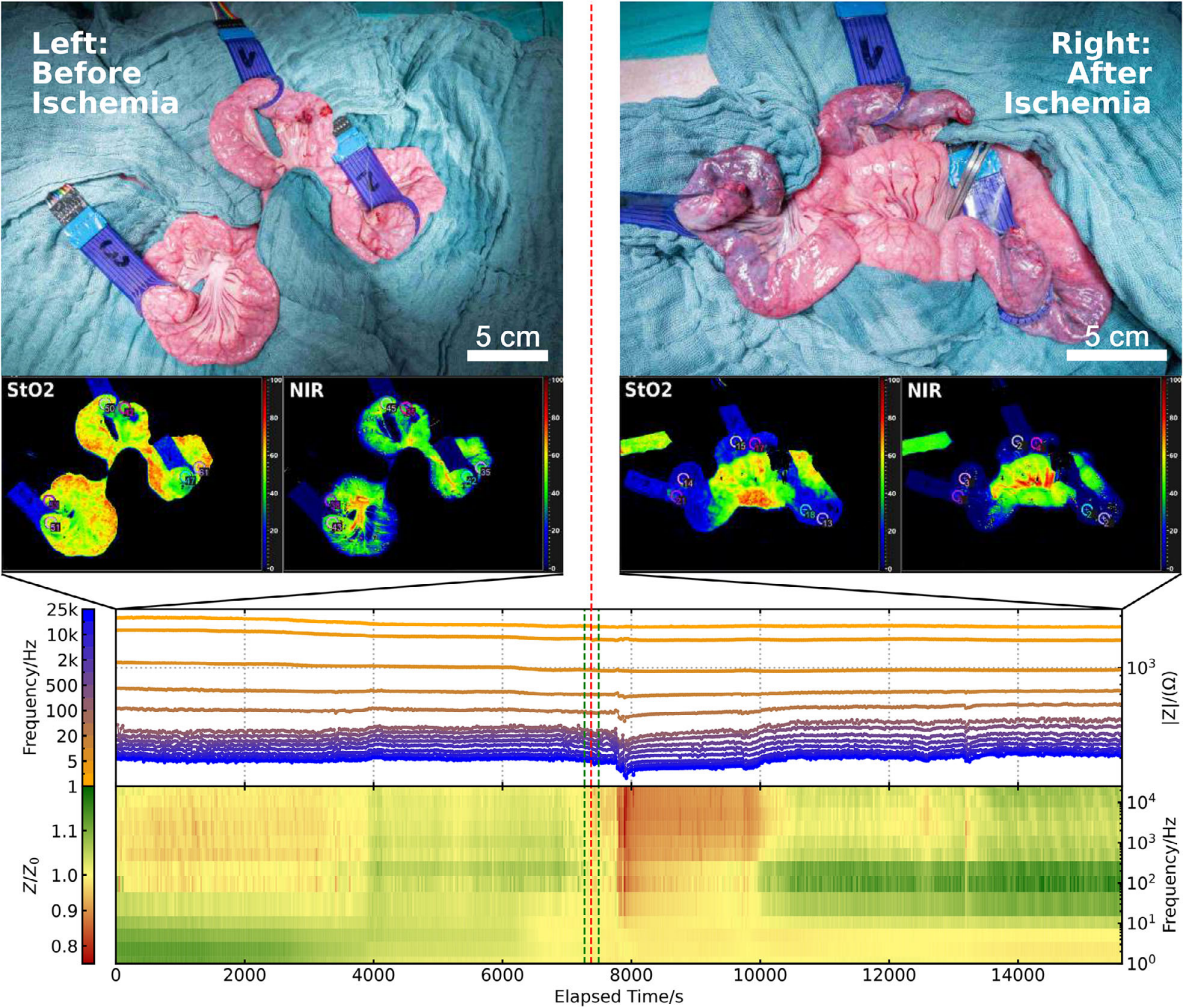
Results: Optical images and hyperspectral data before (left) and after (right) ischemia induction. The segmental ischemia of the anastomoses is visible both in optical and hyperspectral data. Surface tissue oxygen saturation (StO_2_ in %) and near‐infrared perfusion (NIR) have drastically decreased, confirming the induction of ischemia. Below, exemplary impedance data of one electrode pair. The upper plot shows the raw impedance data over time for each recorded frequency. Low frequencies have a more yellow color, high frequencies probing the resistive regime of the impedance have a blue color. The lower plot shows impedance values normalized to the impedance measured at the time of ischemia induction. Here, the normalized impedance is color‐coded in the z‐axis, green indicating an increase and red a drop in tissue impedance. This is done for each frequency (y‐axis) and each time (x‐axis). Green vertical dashed lines indicate start and end points of manipulation; the red vertical dashed line indicates the time of mesenteric vessel ligation. About 10 min after the blood supply stopped, the impedance drops significantly for frequencies above 100 Hz. No other external influence, which could explain this behavior, was observed, indicating a causality of ischemia induction and impedance drop.

**Figure** **2** provides an exemplary overview of one complete measurement set of one sensor. The top row shows optical images before and after ischemia induction (i.e., before starting and after ending the impedance measurements), documenting the visible changes in tissue appearance. Below, the corresponding hyperspectral data are depicted, validating the ischemia induction. In the bottom, the impedance values *Z* measured by our DAQ devices are shown. The top plot shows the raw impedance values over time for each measured frequency. One can clearly see the capacitive electrode behavior, where the impedance is decreasing with frequency. For frequencies higher than 100 Hz, the impedance values become almost constant, indicating that these frequencies preferentially probe the resistive part of the impedance. Below, the same data, normalized to the impedance at the time of ischemia induction, are displayed in a time‐frequency heatmap. Here, frequencies are arranged along the y‐axis, time along the x‐axis, and the normalized impedance values are color‐coded, allowing visual identification of frequency‐dependent changes in tissue properties.

In general, a stable baseline is achieved under optimized conditions. However, the system remains susceptible to occasional signal fluctuations not attributable to physiological changes. In particular, we observe sporadic shifts in impedance during the baseline phase, around 3.5 % at seemingly random points in time, e.g., at 4000 s in the measurement displayed in Figure 2. These changes are not linked to any intentional intervention and occur despite otherwise stable external conditions.

Despite these limitations, the baseline remained sufficiently stable to allow reliable differentiation of subsequent physiological signals. For classification of the baseline, the mean and standard deviation are calculated for each frequency. Noise levels remained routinely below 5 % of the recorded impedance level (compare also **Figure** **4** and Section 3.4).

**Figure 3:**
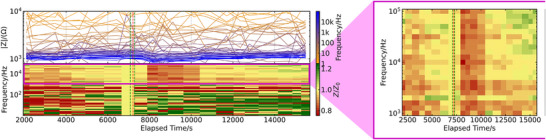
Data recorded with the Autolab PG‐STAT302N potentiostat for setup validation. Compared to the DAQ device, the data is very noisy for low frequencies due to a lower signal amplitude used by the instrument. However, impedance recorded at high frequencies shows a similar behavior to the data presented in Figure 2, where the signal‐to‐noise ratio is sufficiently high. The baseline is relatively stable, with a gradual increase up to ischemia induction, indicated by the red‐dashed line. Here, a pronounced drop in impedance after ∼10 min for frequencies *f*>1 kHz is visible. The signal strength here is around 15 % of the recorded impedance levels. After another 30 min impedance recovers to pre‐ischemia levels, and shows a gradual increase up to the end of the measurement.

**Figure 4:**
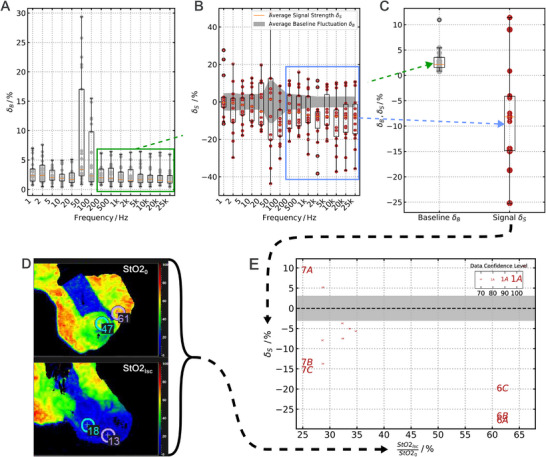
Baseline and signal analysis: For a total of seven anastomoses with three impedance sensors each, the data are assessed and a trust level manually assigned to the sensor (for details see Supporting Information). For trust levels ⩾ 70 %, sensors were considered for a pooled analysis presented here: A) Analysis of temporal baseline fluctuation δ_*B*_; For each frequency, standard deviation of the impedance signal is calculated over a defined pre‐ischemic period, and normalized to the mean impedance of this period. Average baseline fluctuation levels δ_*B*_ routinely stay below 5 %. B) The signal strength δ_*S*_ is extracted from the data for each frequency and compared to the average noise levels from **A** (gray band). For frequencies ⩾ 200 Hz, the average signal strength δ_*S*_ exceeds the average baseline fluctuation δ_*B*_. C) Comparison of averaged fluctuation levels δ_*B*_ for frequencies 200 Hz to 25000 Hz to the average signal strength δ_*S*_ in the same frequency range. D) For each sensor, the oxygen saturation level (StO_2_) is measured to validate the ischemia. The quotient of the ischemic saturation compared to the pre‐ischemic level is calculated. E) Potential correlation of signal strength δ_*S*_ and ischemia strength. A potential correlation is observed, where incomplete ischemia (higher quotients) leads to a stronger drop in impedance. The number and letter markers correspond to the datapoint obtained at the specific anastomosis site by the specific sensor. E.g., 6A corresponds to sensor A of anastomosis 6. The size of the markers indicates the confidence level assigned to the specific sensor.

### 3.2 Ischemia Response

Following the baseline period of 2 h, ischemia is induced as described in Section 2.2.2. The start and end of mechanical manipulation work for ischemia induction are marked by the two green dashed vertical lines in Figure 2. The red dashed vertical line marks the time at which the blood supply to the anastomotic intestinal segment is interrupted. As illustrated in Figure 2, the impedance spectrum stays stable during manipulation, and with a delay of about 10 min, a pronounced drop in impedance is observed across a frequency range from 100 Hz to 25 kHz. This drop reaches a maximum difference of 21 % depending on the frequency and individual sensor, and is clearly visible in both the time‐series plot and in the heatmap.

After the drop in impedance, the values remain at a lower level for about 30 min, after which the impedance recovers to the original level, where it stays for another 50 min. After that, the impedance rises to levels about 7 − 10 % higher than the impedance at the time of ischemia induction. As the baseline noise for this specific sensor is only around 1 %, we consider this a significant increase in impedance.

It should be noted that some sensors exhibit a different signal behavior, including a moderate increase in impedance over time, without an initial rapid drop in impedance. Of the sensors considered for pooled data analysis (see Section 3.4) 85 % of the sensors show a decrease in impedance, while 15 % exhibit an increase in impedance. All data for all anastomoses are depicted in the Supporting Information, Figures S3–S9.

Hyperspectral imaging is performed at the start and the end of the impedance measuring period to validate the presence of ischemia. Both StO_2_ and NIR show a significant decrease following blood vessel blockage across the majority of experiments (see Figure 2, middle row). This serves as a physiological confirmation of successful ischemia induction.

Notably, for one anastomosis, only a partial ischemia was induced, as backed by hyperspectral data. Interestingly, this specific anastomosis showed the strongest impedance response, suggesting that the impedance signal might respond stronger to an incomplete ischemia (see Section 3.4).

### 3.3 High‐Precision Validation

As already outlined, one sensor electrode pair of one selected anastomosis was measured using the Autolab PG‐STAT302N potentiostat to validate our custom DAQ device measurement setup. The recorded data are shown in **Figure** **3**. Here, the same figure structure as for Figure 2 applies; on top, the raw impedance data for each frequency, on the bottom, the normalized impedance over time in a heatmap. Despite the differences in hardware and signal processing, the resulting impedance values for higher frequencies, which probe the resistive part of the impedance (e.g., *f* > 1 kHz) are of comparable magnitude to those measured with the DAQ‐device‐based method. It should be noted that the exact measured impedance can and does vary between individual anastomoses due to imperfections in the production process (i.e., different capacities of the electrodes), and in the implantation process (i.e., different electrode‐tissue coupling). At lower frequencies, the Autolab data show more pronounced noise, likely due to the reduced excitation voltage used by the instrument, resulting in a lower signal‐to‐noise ratio, and thus more noisy data. Nevertheless, the measurement clearly captures a characteristic drop in impedance following ischemia onset.

### 3.4 Pooled Data Analysis

To evaluate the consistency and diagnostic potential of the impedance response across different experiments, we perform a pooled analysis incorporating data from up to seven individual anastomoses. First, all sensor data are manually reviewed, and a qualitative confidence rating is assigned based on baseline stability and measurement artifacts (for details, see Supporting Information Sections S3.1 and S3.2, Figures S3–S9). Only sensors with a confidence level ⩾70 % are included in the pooled evaluation.

In a first step, the temporal signal fluctuation of the baseline δ_*B*_ is analyzed by calculating the standard deviation Δ*Z*_*B*_ of the impedance signal for each frequency over a defined manually assigned pre‐ischemic reference period. This value is normalized to the mean impedance $\bar{Z_{0}}$, yielding a frequency‐resolved noise profile. On average, the relative noise level remained below 5 %, indicating stable measurement conditions across most frequencies, as seen in Figure 4A.

Next, the signal strength δ_*S*_ is extracted from each dataset. First, it is determined if impedance drops or rises after ischemia. Depending on this, the minimum (drop) or maximum (rise) impedance value Δ*Z*_*S*_ is extracted for each frequency. This impedance value is compared to the average baseline level $\bar{Z_{0}}$ determined before. The frequency‐dependent signal strength δ_*S*_ is then compared to the previously determined baseline fluctuation band δ_*B*_ (see Figure 4B). For frequencies higher than 200 Hz, the average signal strength exceeds the average baseline fluctuation levels.

Figure 4C summarizes this behavior. Here, the average baseline fluctuation and signal strengths for frequencies between 200 Hz and 25 kHz across all qualifying sensors (*N* = 13) are calculated and compared. An average impedance decrease of 10 % is observed, with most observed signals exceeding the average noise level, both for impedance drops and increases.

Finally, we correlate the impedance signal strengths δ_*S*_ with the degree of ischemia, as quantified via hyperspectral imaging. For this, the ratio of post‐ischemic to baseline tissue oxygen saturation $\frac{{\text{StO}}_{2,0}}{{\text{StO}}_{2,\text{Isc}}}$ ischemia induction is calculated (see Figure 4D). Ratios below 100 % indicate a reduction in oxygenation (lower values indicate stronger ischemia), while ratios above 100 % indicate an improvement. Interestingly, we observe a higher signal strength for a weaker ischemia, as seen in Figure 4E.

## 4 Discussion

### 4.1 Interpretation of Bioimpedance Signals

**Figure 5:**
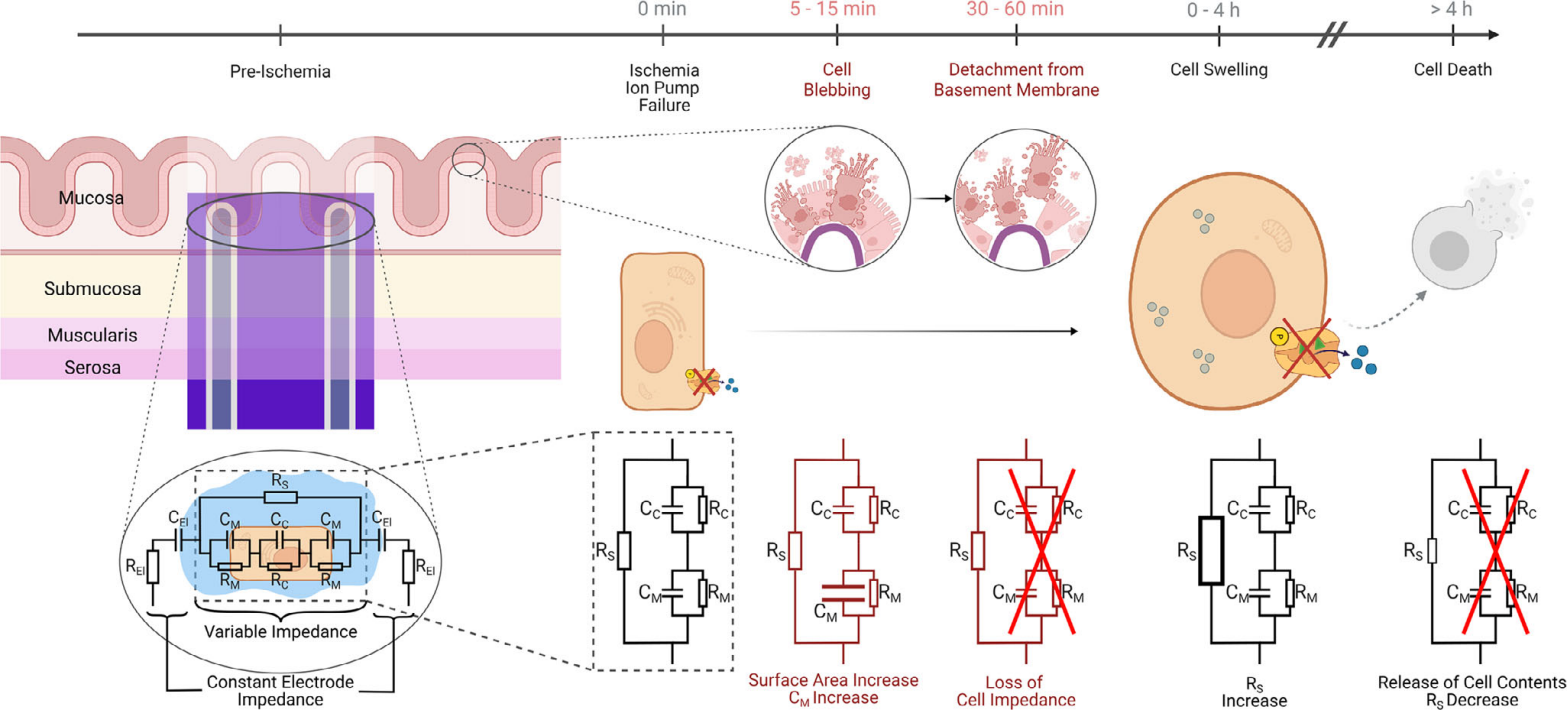
Hypothesized pathophysiology of the observed impedance signals. The leftmost part illustrates the sensor's positioning within the intestinal tissue. Here, the sensor is in contact with all tissue layers. Depicted below is a simplified proposed equivalent circuit of the sensor interfacing with the tissue: The electrodes are represented by a series RC element (*R*_El_, *C*_El_). These electrodes are in contact with the ionic extracellular environment, which is represented by a series resistor *R*_S_. The cells are represented by a series of parallel RC elements, representing both the cell membrane's (*C*_M_, *R*_M_) and the cell content's (*C*_C_, *R*_C_) capacitance and resistance. Both the impedance of the extra‐ and intracellular environment will vary in the experiment, while we assume the electrode's impedance to be constant. The evolution of this variable impedance is illustrated here for the various physiological processes occurring in the measurements, as described in Sections 4.2 and 4.2.1. After ischemia induction, ion pumps fail, leading to a steady increase in intracellular sodium levels. In the mucosal layer, cell blebbing occurs, which leads to an increase in *C*_M_, resulting in a drop in impedance. After 30 min to 60 min, these mucosal cells detach from the basement membrane, resulting in a removal of the cell impedance part and a subsequent recovery and increase of the impedance. In all cell layers, starting from the time of ion pump failure, cells start to swell, leading to a reduction of available conducting material in the extracellular space, resulting in an increase in *R*_S_, and the resulting increase in impedance. After >4 h (not measured in the experiment), cells ultimately die, releasing their contents into the extracellular environment. This increases the local ion concentrations and results in a decrease in series resistance *R*_S_.

Bioimpedance describes the complex electrical resistance of tissue as a function of small signal excitation. This small signal excitation ensures that no alterations to physiological processes take place. However, to interpret the measured impedance signals, it is crucial to understand the electrical properties of the tissue. The interaction between the electrodes and the biological tissue can be conceptualized using a simplified equivalent circuit model, depicted in **Figure** **5**. In this experiment, the impedance sensing electrodes penetrate the whole intestinal wall and are in contact with all intestinal cell layers (Mucosa, Submucosa, Muscularis, and Serosa). Consequently, processes in each layer may influence the observed impedance signal. The simplified equivalent circuit consists of the following items:

- The electrodes, modeled by a series RC element (*R*_El_, *C*_El_),
- The extracellular space, modeled by a series resistor (*R*_S_), reflecting the conductivity of the ionic environment,
- The cells, modeled by two serial parallel RC elements: the membrane (*R*_M_, *C*_M_), and the impedance of all cell contents (modeled by *R*_C_, *C*_C_)

Here, the electrode impedance is assumed to be constant, and changes in the observed impedance stem mainly from changes in the extra‐ and intracellular environment.^[^ ^23^, ^24^, ^25^, ^26^, ^27^ ^]^

The contribution of each element in this circuit depends on the frequency of the applied signal. At low frequencies (1 kHz to 10 kHz), the capacitive properties of intact cell membranes dominate the measured impedance, and current flows mainly through the extracellular space. At intermediate to high frequencies (>10 kHz), the capacitive contribution of the cell membranes to the observed impedance decreases, allowing current to pass through the cell membrane and probe the intracellular components. In this range, both extracellular and intracellular processes affect the observed impedance.^[^ ^17^, ^28^, ^29^, ^30^, ^31^ ^]^

Changes in the cells caused by ischemia consequently lead to changes in the cells' electrical properties, which should be reflected in the observed impedance.^[^ ^23^, ^24^ ^]^ In the following section, pathophysiological mechanisms explaining the observed impedance changes are proposed.

### 4.2 Pathophysiology of Observed Signals

Following ischemia induction, oxygen and glucose deprivation lead to ATP depletion. Additionally, gap junctions close under ischemia in an attempt to limit the spread of apoptotic signals, accelerating ATP depletion and oxygen consumption.^[^ ^32^ ^]^ This ATP deficiency impairs the Na‐K‐ATPase as well as various other ion pumps. As ionic gradients collapse, intracellular sodium accumulates, leading to water influx into the cell and cell edema.^[^ ^15^, ^33^, ^34^, ^35^ ^]^ This cell swelling reduces extracellular volume and compresses the intracellular space.^[^ ^24^, ^36^ ^]^ Previous studies have related this to an increase in tissue impedance over an estimated period of 3 h to 4 h. In the equivalent circuit presented in Figure 5, this would lead to an increased *R*_S_, as indicated in the equivalent circuit during cell swelling. These well‐documented mechanisms are consistent with our observed gradual post‐ischemic impedance rise approximately 30 min to 60 min after ischemia induction. Following this interval of cell swelling, irreversible cell death occurs, characterized by rupture of cell membranes.^[^ ^37^ ^]^ This leads to the release of intracellular contents into the extracellular space, increasing ionic concentrations, lowering the extracellular resistance *R*_S_. However, these changes were not represented within the chosen timeframe.

Before the post‐ischemic impedance rise, we managed to observe a previously uncharacterized impedance drop within 8 min to 15 min following ischemia induction, followed by an increase 30 min to 60 min after the impedance drop, enabled through the high temporal resolution and the precise intra‐anastomotic positioning of the sensors. To our knowledge, only one study by Hou et al.^[^ ^37^ ^]^ reported a similar initial decrease in impedance. In their study, electrodes were applied to the serosal surface of resected segments of human small intestine immediately post‐resection, and impedance was monitored once every hour up to 10 h. In the first hour, impedance decreased, followed by an increase of impedance up to 4 h. The authors attributed the early impedance drop to environmental changes related to the repositioning of the tissue from the intraabdominal cavity to a thermal box. In contrast to this, our in vivo measurements are conducted under stable physiological conditions, without environmental perturbation, and yet an initial decrease of impedance is observed. The reproducibility of the early impedance drop across multiple measurements (both this study and Hou et al. [37]) suggests that it may reflect an underlying physiological process rather than a measurement artifact. In the following section, we present a hypothesis for the physiological origins of this early signal.

#### 4.2.1 Early Impedance Drop as Hyperacute Ischemia Marker

While the reproducibility and timing of the observed early impedance drop suggest a physiological origin, its exact underlying physiological causes remain highly speculative. In the absence of direct histological validation, the following interpretation must be regarded strictly as a hypothesis. Nonetheless, we propose a plausible explanation for this phenomenon based on known patterns of early ischemic mucosal injury and previously reported cellular mechanisms.

The intra‐anastomotic positioning of our sensor allows for high temporal and spatial resolution, enabling the detection of subtle, localized changes. It is well established that the intestinal mucosa, particularly the epithelial lining of the villus tips, is highly susceptible to short‐term ischemia.^[^ ^38^ ^]^ Based on this, we hypothesize that the early impedance decrease observed approximately 8 min to 15 min, may reflect initial mucosa responses to ischemia. One process, consistent with this timeframe, is the onset of cell blebbing. It involves the formation of protrusions on the cell membrane surface, resulting in an increased total membrane surface. Since the cell membranes contribute to the capacitive component of the tissue impedance, an increase in membrane surface area leads to an increased capacitance (*C*_M_ in Figure 5) and thus a measurable decrease in overall electrical impedance.^[^ ^37^ ^]^

Approximately 30 min after ischemia induction, the decrease in impedance starts recovering to pre‐ischemia levels. This transition aligns with previous histological studies demonstrating the formation of Gruenhagen's spaces, subepithelial clefts indicating detachment of mucosal epithelial cells from the basement membrane, within 15 min to 30 min. Around 60 min post‐ischemia, epithelial sloughing becomes apparent,^[^ ^39^ ^]^ marking the end of the early mucosal phase and the beginning of the more advanced, transmural tissue ischemia, represented by a slow but steady increase in impedance.

### 4.3 Data Quality

Ensuring high data quality is essential for the interpretation and reliability of bioimpedance measurements, particularly in dynamic in vivo experiments. This section addresses how specific technical and procedural factors influence the recorded signals and discusses potential artifacts that may affect data quality.

#### 4.3.1 Influence of Measurement Setup

The DAQ‐based measurement system (see Section 2.2.3) enables parallel, real‐time impedance spectroscopy at multiple sites around the anastomosis. However, several hardware‐related factors may affect signal precision and stability, particularly under in vivo conditions.

One factor is the use of a fixed 2.2 kΩ reference resistor. If tissue impedance deviates significantly from this value, the limited resolution of the DAQ device reduces measurement accuracy and Signal‐to‐Noise ratio. This resistor is chosen to match typical impedance values recorded in the anastomosis to minimize this effect. Comparative laboratory experiments with the PG‐STAT302N show no relevant spectral differences within the frequency range in which the impedance signals are observed.

Commercial FPC connectors and standard jumper cables link sensors to the DAQ. While these connectors introduce contact resistance and are sensitive to movement, securing the pads with epoxy glue prevented instability and impedance changes due to movement. Minor impedance shifts (≪1 % of *Z*_0_) remained when bending the printed silver ink, due to the inherent ink properties. Long cable lengths of >1 m enabled practical placement, but non‐existent shielding makes the cables susceptible to electromagnetic noise. However, most noise observed originated from poorly grounded USB ports of the measurement laptop connected to mains power.

Overall, the measurement system allows for a high spatial and temporal resolution in vivo. While the obtained data quality is already satisfactory, the factors mentioned above highlight important technical challenges to be addressed in future iterations of the setup.

#### 4.3.2 Measurement Artifacts

Due to the sensitivity of in vivo bioimpedance measurements to both physiological and technical influences, various artifacts are observed. One recurring issue are spontaneous impedance shifts of around 3.5 % without intentional intervention (compare data at 4000 s in Figure 2). These often coincided with changes in signal noise (Figure S8, CH B, Supporting Information), suggesting electromagnetic interferences, possibly induced by insufficiently isolated mains‐powered equipment (e.g, 50 Hz noise from the laptop power supply).

Pronounced jumps in impedance are mainly associated with strong mechanical manipulation of the intestine (see Figures S2–S4, Supporting Information). As mechanical instability at the FPC connector is excluded, these jumps might arise from minor changes in electrode‐tissue coupling or structural damage to the sensor electrodes due to strain.

Additional artifacts include brief impedance drops following application of electrolyte solution (Figure S3, Supporting Information), with quick recovery to previous baseline levels.

Furthermore, potential crosstalk between sensor channels, due to the electrolytic abdominal environment, needs to be considered. Especially at low frequencies, which penetrate tissue more deeply, some sensors pick up the excitation signals of the PG‐STAT302N, leading to periodically increased noise (see Figure S9, Supporting Information).

While such disturbances are infrequent and clearly identifiable, they emphasize the need for rigorous signal interpretation. A more detailed discussion is provided in the Supporting Information, Figures S3‐S9.

#### 4.3.3 Data Variability

As mentioned in Section 3.2, the predominantly seen signal is a sudden decrease in impedance for high frequencies, followed by a gradual increase up to the end of the measurement. While this distinct drop in impedance is observed in at least one sensor for 5 of the 7 anastomoses following ischemia induction, some sensors exhibited a different signal behavior, including a moderate increase in impedance over time, without a prior drop in impedance. Although this is not the prevailing trend, it needs to be considered in the interpretation of the results.

Several factors may contribute to these variations. A technical explanation could be the spatial positioning of the sensors within the abdominal cavity. Sensors located at a lower gravitational point could observe a higher impedance, due to accumulating intraperitoneal fluids, which could attenuate the measurable impedance drop, or even cause an apparent increase in impedance. Additionally, ischemia‐induced cellular and vascular responses might lead to local shifts in electrolyte concentration. In particular, the release of interstitial fluid or dilution of extracellular electrolytes could increase the apparent tissue impedance locally, resulting in an upward trend of the recorded impedance.

Another factor could be slight differences in the positioning of the electrodes and the electrode‐tissue coupling. Sensors not directly coupled to the mucosal layer may fail to detect the hypothesized early epithelial response, such as membrane blebbing and the associated rise in capacitive coupling. Instead, only the later‐stage ischemic changes characterized by edema, cell swelling, and necrosis, are seen, resulting in an observed increase in impedance. Together, these factors underscore the complexity of in vivo impedance spectroscopy and the influence of both physiological and geometrical factors on signal behavior.

Despite the inherent challenge and the described variability of in vivo impedance measurements, we place high confidence in the observed ischemia‐related signal drop, which is presented in Figures 2, S8, and S9. This assessment is based on extensive preliminary experimentation, based on which the presented experimental protocol is developed. In particular, special care is taken to minimize movements and manipulation of the bowel after sensor placement and especially during ischemia induction, as strong movements can alter tissue‐electrode coupling and thus affect the observed impedance. During and after the ischemia induction, no external factors such as temperature changes, mechanical disturbance, or additional fluids are introduced into the anastomosis, which could explain the observed signal behavior.

### 4.4 Correlation with Ischemia Severity

Interestingly enough, the initial decrease seems to correlate with a higher StO_2_ difference, equaling a reduced perfusion (in contrast to a complete occlusion of the supplying blood vessels) and thus partial ischemia. Our results indicate that a less severe ischemia leads to a distinct decrease in impedance. While the exact underlying physiological mechanisms leading to this finding remain unclear, we hypothesize that a short‐term, functional reactive hyperemia might play a role.

When inducing a sudden ischemia, existing collateral circulation may functionally mimic the physiological response of reactive hyperemia. This can also be referred to as “responsive”^[^ ^40^ ^]^ or “low perfusion”^[^ ^41^ ^]^ hyperemia. Following tissue hypoxia, local autoregulatory mechanisms, induced by accumulation of metabolic products (potassium, adenosine due to increased ATP degradation, lactate, CO_2_), as well as endothelium‐mediated NO release, promote local vasodilatation.^[^ ^40^, ^42^, ^43^ ^]^

We hypothesize that these mechanisms may also affect patent vessels (e.g., marginal arcades), leading to an enhanced collateral perfusion. This results in an acute, short‐term fluid transfer into hypoxic tissue, which ultimately causes a decrease of tissue impedance.^[^ ^44^, ^45^ ^]^

Such explanations should be approached with caution, since this observation has not been made so far. Nevertheless, this observation constitutes a recurring pattern, indicating a causal relationship.

### 4.5 Relevance in the Context of Current Research

As previously stated, the timing of diagnosis of AL is a critical determinant of patient outcome. This highlights the urgent need for novel diagnostic approaches that allow for earlier and more precise detection. Considering recent advances in biomedical engineering, the use of real‐time monitoring technologies offers significant potential to improve postoperative care. Traditional approaches that rely on intermittent clinical assessments, typically performed once or twice daily, may fail to detect early signs of complications. In contrast, continuous monitoring can provide immediate feedback, enabling earlier identification of adverse events. The integration of such technologies into routine clinical practice could enhance patient safety, reduce intervention delays, and ultimately lead to improved clinical outcomes. Recent efforts to enable an earlier diagnosis of AL have focused on sensor‐based technologies. Jessernig et al. developed a sensor array for continuous monitoring of digestive enzymes in surgical drain fluids.^[^ ^46^ ^]^ Similarly, Huynh et al. proposed a device for measuring pH levels in the drain fluids to identify early biochemical signs of leakage.^[^ ^47^ ^]^ Exero Medical's xBar system, currently undergoing clinical trials, based on the studies of Ben‐David et al.,^[^ ^48^ ^]^ uses perianastomotically placed electrodes and electrodes placed in the surgical drain, while monitoring the impedance. In their study, a colotomy was performed to simulate AL.

The growing interest in such technologies underscores the clinical need for faster and more reliable diagnostic methods. All these approaches, however, inherently rely on the externalization of intraperitoneal fluids, detecting leakages only after bowel perforation has occurred and enteric contents have leaked, potentially delaying diagnosis and therapeutic interventions. In most cases, they can not capture the ischemic cascade before dehiscence of the anastomosis occurs. Compared to current diagnostic systems, which rely on detecting the extravasation of bowel contents, our intra‐anastomotic sensor device could offer several advantages. Most notably, it allows for continuous and localized monitoring of the tissue status directly at the anastomotic site, which is the most susceptible to failure. This potentially enables earlier detection of cellular injury before irreversible damage occurs. The use of a resorbable PDO substrate ensures bioresorbability and compatibility with established surgical techniques, laying the foundation for a fully resorbable system, eliminating the need for device retrieval surgeries. The circumferential sensor configuration permits high spatial resolution and allows for potential internal cross‐validation across multiple sensing sites.

### 4.6 Study Limitations and Challenges

Despite our promising results, several limitations of this study must be acknowledged. Most notably, the experiments were conducted on porcine intestine, rather than human. Particularly in the intestinal tract, pigs exhibit many anatomical and physiological similarities to humans, highlighting the translational potential of the porcine model.^[^ ^49^, ^50^, ^51^ ^]^

The experimental setup was limited to anesthetized animals. Future studies will need to assess the influence of physiological conditions in awake, mobile animals on bioimpedance. While we only performed impedance measurements for approximately 4 h, the electrodes and sensors themselves are able to persist considerably longer. Laboratory degradation tests of encapsulated silver resistors showed a lifetime of >20 d while immersed in minimal essential medium (MEM) as a proxy for the complex electrolytic body environment (see also Supplementary Information Section S4, Figure S10). It should be noted here that with the current measurement setup, only stationary measurements on an open abdomen can be realized. Measurements significantly longer than 4 h, e.g., over days, can not be realized on anesthesized pigs due to animal welfare regulations and would require a portable and/or implantable measuring system.

Ischemia, a main contributor to AL, was deliberately induced in this model. It is important to recognize that AL can occur in the absence of ischemia and, conversely, may not develop despite compromised blood flow to the anastomotic site. *It should be noted that, although arterial ischemia was induced in this experimental setting, delayed venous ischemia may also occur in clinical cases. Owing to the design and objectives of the present study, we focused on the rapidly developing arterial form. Nevertheless, by continuously monitoring the anastomotic site, we hypothesize that venous ischemia would produce a comparable impedance signature over time and could therefore be detected by our sensor. It is important to emphasize that the sensor device is not intended to replace existing interventional therapies but rather to serve as an assistive tool, supporting the surgeon in making timely decisions regarding therapeutic intervention. In this context, early detection of ischemic changes could be particularly valuable for identifying cases of partial ischemia or impending anastomotic leakage, where prompt initiation of vacuum sponge therapy may substantially improve clinical outcomes*.^[^ ^52^, ^53^, ^54^ ^]^

To enhance clinical relevance, future experiments should also incorporate sensor placement within esophageal and rectal anastomoses, simulating the use of circular staplers in surgical practice. Hereby addressing also the larger size of used circular staplers in human operations. In the present study, the small intestine was utilized to increase the number of anastomoses per animal, thereby reducing overall animal use in accordance with ethical guidelines.

The sensor geometry has been optimized for use with standard 21 Ethicon circular staplers; however, the manufacturing process, based on screen printing technology, allows for facile modification of sensor components to different implantation sites and stapler sizes.

Additionally, the DAQ device‐based measurement setup requires a stationary PC. This limits data recording to the duration of the surgery. As these devices are inherently non‐implantable, long‐term measurements are not feasible with this setup. Furthermore, cables and connectors add potential for artifact introductions. Future experiments should include a custom integrated circuit and PCB with the task of recording impedance spectra during a temporary implantation period after anastomosis creation.

It should be noted here that while our sensors are sensitive to ischemia induction, we have not thoroughly investigated the specificity. First data shows a sensitivity to other processes outside of the anastomotic site (e.g., addition of electrolyte solutions into the abdominal region, see Figure S3, Supporting Information).

In the absence of fully automated quality control or pattern recognition, data quality in this study was primarily assessed through manual inspection of the recorded impedance signals. Data quality assessment was also heavily based on the data recorded by the high‐precision PG‐STAT302N, which we consider to be the true signal. However, this process inherently involves a degree of subjectivity, as decisions regarding inclusion or exclusion are often based on visual judgment and prior experience. To reduce potential observer bias in future studies, the implementation of blinded data assessment protocols and standardized signal quality metrics will be essential. These would enable more objective and reproducible classification of data quality, particularly in studies involving larger datasets.

The sensing electrodes are not yet resorbable in this iteration of the experiment, with the screen‐printable silver ink the only non‐degradable component. However, as the need for transient and implantable electronics will increase, more fully resorbable inks will appear for use. Previous works have shown that zinc can be integrated in a resorbable polymeric binder to create a screen printable, fully resorbable ink.^[^ ^55^, ^56^, ^57^ ^]^ In our own recent study,^[^ ^18^ ^]^ a zinc ink based on polycaprolactone binders demonstrated good adhesion on PDO substrates and could, in principle, replace the silver ink used in this experiment. Due to the lower electrode capacitance of such zinc electrodes, higher mesaurement frequencies are required to access the same resistive information probed by the Ag/PEDOT:PSS electrodes used in this study, motivating future adaptations of readout hardware.

From a translational perspective, two pathways for integration into clinical practice can be envisioned. A fully resorbable and wireless approach would eliminate retrieval procedures altogether. Early steps toward wireless communication have been demonstrated with fully resorbable resonant circuits on PDO.^[^ ^18^ ^]^ However, this route potentially faces challenges such as the lack of resorbable active electronics or signal attenuation in tissue. Alternatively, a wired approach via the surgical drain offers a short‐term solution. The resorbable sensor consisting of PDO substrates and electrodes would remain inside the anastomosis, while a small non‐resorbable readout unit is connected externally through the drain. Once the drain is removed in a standard clinical procedure, the readout unit can be retrieved without additional procedures required.

While the present study employs a simple two‐electrode configuration, this design primarily serves as a proof of concept for intra‐anastomotic sensing. The screen‐printing sensor‐fabrication process, however, allows for the implementation of more advanced electrode architectures. Future iterations may incorporate three‐ or four‐electrode setups to enable more sophisticated impedance measurements, potentially reducing motion‐related artifacts. Moreover, the same platform can be adapted for electrochemical sensing by integrating functionalized electrodes, or even organic electrochemical transistors (OECTs), enabling amperometric or voltammetric sensing of specific physiological markers (e.g., lactate^[^ ^58^ ^]^) directly at the anastomotic site.

## 5 Conclusion

In this feasibility study, we demonstrate for the first time the use of implantable, organic bioimpedance sensors for real‐time intra‐anastomotic monitoring of ischemia‐related tissue changes in vivo. The sensor device, based on PDO, screen‐printed silver, and PEDOT:PSS, shows promising biocompatibility and has proven mechanical stability both during implantation and in the critical postoperative period. While full resorbability has not yet been achieved, material optimization efforts are ongoing toward a fully degradable sensor platform.

Our data confirm that bioimpedance spectroscopy enables real‐time detection of physiological changes associated with early ischemic injury. In several experiments, we observe a recurring impedance signature, typically characterized by an initial drop followed by a gradual increase. This may provide a basis for future pattern‐recognition algorithms aimed at early detection of ischemic changes. However, this pattern is not observed consistently across all datasets, and further investigations are required to clarify the sources of variability and refine the underlying biological model.

While the results are encouraging, they must be interpreted with caution given the limited sample size, the absence of blinded analysis, and the experimental constraints inherent to preclinical studies. Nonetheless, our findings provide both a technological and conceptual foundation for novel intra‐anastomotic measurement devices, as well as a set of preliminary observations that motivate further in vivo studies and the implementation of real‐time data analysis algorithms. Despite aforementioned challenges, this approach could support the development of a fully resorbable, clinically applicable monitoring system that enables early detection of anastomotic failure and potentially facilitates timely intervention. Ultimately, we were able to successfully detect ischemia‐related changes at the anastomotic site by using an innovative, intra‐anastomotically placed, resorbable sensor device.

## Author Contributions

D.W. and F.J. contributed equally to this work and share first authorship. Both authors are permitted to list their names first in curricula vitae and other academic contexts, provided equal contribution is indicated. C.S. and K.L. formulated and designed the study equally, provided theoretical guidance and revised the manuscript. D.W., F.J., D.F., J.H., S.H., R.K., and H.K. performed the experiments ‐ separated in fields of expertise by their respective affiliations. D.W. organized under guidance of S.H. the in vivo experiments. F.J. produced under guidance of K.L. and H.K. the sensor device and prepared the data acquisition systems. H.K. provided technical guidance. A.Z. performed anesthesia. E.G. and B.V. provided expertise for the in vivo experiments. F.J., assisted by R.K., performed the data analysis. F.J., D.W., J.H., and R.K. wrote the manuscript. All authors critically reviewed the manuscript. Funded by the “Deutsche Forschungsgemeinschaft” (DFG), project “FAVORS” 461264398 Funded by the European regional development fund, reference number: EXF‐25‐2010

## Conflict of Interest

Authors D.W., F.J., D.F., E.G., H.K., J.H., S.H., C.S., K.L. are co‐inventors on a pending patent application (DE10 2024 137 023.8) covering aspects of the technology described in this article.

## Supporting information



Supporting Information

## Data Availability

The data that support the findings of this study are available from the corresponding author upon reasonable request.
