# Supplementary material for: Real‐Time In Vivo Monitoring of Anastomotic Intestinal Ischemia Using Implantable Resorbable Organic Sensors
Source: Adv Sci (Weinh). 2025 Dec 3;13(16):e14507. doi: 10.1002/advs.202514507 (PMC13042606; doi:10.1002/advs.202514507)
Supplement: Supplementary file 1 — Supporting Information [file ADVS-13-e14507-s001.pdf]

Supplementary Information

Real-Time *In Vivo* Monitoring of Anastomotic Intestinal Ischemia Using Implantable Resorbable Organic Sensors

Finn Jaekel, Dennis Wahl, Julia Henne, Richard Kantelberg, Daniel C. Freund, Eberhard Grambow, Brigitte Vollmar, Amelie R. Zitzmann, Hans Kleemann, Jochen Hampe, Sebastian Hinz, Clemens Schafmayer, Karl Leo

S1 Introduction to Supplementary Information

To improve clarity and concision of the main manuscript, detailed descriptions of the sensor design and fabrication process and the experimental procedure have been relocated to the Supplementary Information. The following text sections provide additional methodological detail and context regarding the design and implementation of the presented sensor system, including material-specific considerations and data acquisition protocols.

S2.1 Sensor Design

S2.1.1 Substrate Geometry

The shape and geometry of the substrate are tailored to be stapled by a 21 mm ETHICON™ Circular Stapler (Ethicon, Norderstedt, Germany), which is used to fire the anastomoses. An outer ring with an inner diameter of 21.38 mm and an outer diameter of 28.88 mm is designed to clear the anvil diameter and provide sufficient area on the outer ring to fit all sensors and supply lines (see Figure S1). The outer ring is connected to an inner ring with an outer diameter of 10 mm by a total of 12 spokes, with an angular distance between each spoke of 30◦. Every 90◦ a spoke with a width of 2 mm is placed, all other spokes are 0.6 mm wide. On the inner ring, a circular cutout of 6.4 mm radius is placed to account for the shaft of the circular stapler. A rectangular cutout of size 6.6 mm × 5 mm is added in the center, so that the ring fits the widest parts of the shaft. The center ring enables reproducible positioning and alignment of the sensor on the stapler and is removed during stapling. A contact pad with a width of 23.24 mm and a length of 52 mm is cut from the same PDO sheet. Its width is chosen to match an 8-pin Amphenol Clincher™ FPC connector. The contact pad is longer than required for the FPC connector to spatially separate the connector from the anastomotic site, thereby reducing potential interference from local fluids or mechanical stress.

S2.1.2 Electrode Configuration

The electrode configuration is primarily defined and constrained by the specifications of the impedance measurement setup. In the present setup, we use a MCC USB-1208HS-4AO High-Speed USB Data Acquisition (DAQ) Device (Digilent), which provides four analog outputs and eight analog inputs. As each impedance measurement requires one output channel to inject current and two contacts (input and return path), the system supports up to four impedance measurements per device. Consequently, the available number of electrode terminals is limited to eight. Out of these eight contacts, two are reserved for a reference

resistance structure. This resistive structure is implemented as a meandering conductor with a line width of 300 μm and a minimum distance of 300 μm positioned on the distal end of the sensor ring and connected to the two outermost pins.
The remaining six electrodes are arranged in pairs on three of the four 2 mm wide spokes on the sensor. Each electrode is 500 μm wide and extends approximately 4 mm radially into the spoke. An inter-electrode gap of 800μm is chosen to maximize the measurable impedance magnitude on one wide spoke, thereby enhancing the signal-to-noise ratio and facilitating more reliable detection with the given acquisition system (see also Figure S1b and S1c) The chosen spacing also makes efficient use of the available surface area on the sensor spokes while maintaining sufficient edge clearance to accommodate fabrication tolerances and minimize the risk of structural defects. Electrodes are not extended further towards the center of the sensor to prevent mechanical damage during stapling, as the central ring of the substrate is cut by the stapler knife during deployment, and could otherwise pull on and detach the exposed electrodes.
In principle, the number of measurement positions could be increased by incorporating multiple DAQ devices per implant. However, due to increased complexity, synchronization requirements, and higher cost, a single-device configuration is chosen for this prototype implementation.

S2.2.3 Sensor Fabrication

The sensors are fabricated by screen printing conductive structures onto 78 mm × 35 mm sized PDO sheets, cut out of a 380 mm×210 mm sheet of extruded 150 μm thick PDO, supplied by Ethicon Inc. (Norderstedt, Germany). In a first step, a silver-based conductive ink (DM-SIP-3060, Dycotec Materials) is printed using a 165-31 mesh screen to define all contact pads, measurement electrodes, the reference resistor structure, and interconnecting traces. Here, silver is selected due to its good biocompatibility, chemical stability, and the widespread availability of low-temperature printable ink formulations compatible with low-melting-point substrate materials. The samples are dried in a convection oven at 50 ◦C. Next, a layer of PEDOT:PSS (Sigma Aldrich, Product Number 768650) is printed using a 165-31 mesh screen to cover only the measuring electrodes on the wider spokes. PEDOT:PSS improves electrode-tissue coupling by increasing the electrode’s capacity, thus reducing the interfacial impedance. This material combination supports both impedance spectroscopy and offers the potential for future implementation of amperometric sensing through additional functionalization of one or more electrodes. The PEDOT:PSS layer is likewise dried at 50 ◦C. To stabilize the printed structures in electrolytic environments, a second PDO layer is laminated on top of the substrate as an encapsulation layer, motivated by earlier findings indicating delamination of conductive traces under physiological conditions [18]. The encapsulation layer includes cutouts to expose the central sensor ring area in contact with the tissue and the contact pad areas. Lamination is performed by sandwiching both PDO layers between two PET sheets and feeding them through a pouch laminator (RM SKY 325 R6) at 120 ◦C. This process ensures bonding without exceeding the thermal budget of the PDO substrate or damaging the printed structures [18]. Following lamination, the final sensor geometry, including spokes, ring openings, and alignment features, is defined by laser cutting. The completed sensors are contacted using an 8-pin Amphenol Clincher FPC connector, which interfaces with standard jumper cables. To mechanically seal and electrically isolate the connector region, the entire assembly is encapsulated using a two-component epoxy adhesive (UHU Plus schnellfest). This step

further reduces the influence of local fluids and motion artifacts on the measured signal. The full fabrication process is depicted in the main manuscript Figure 1.

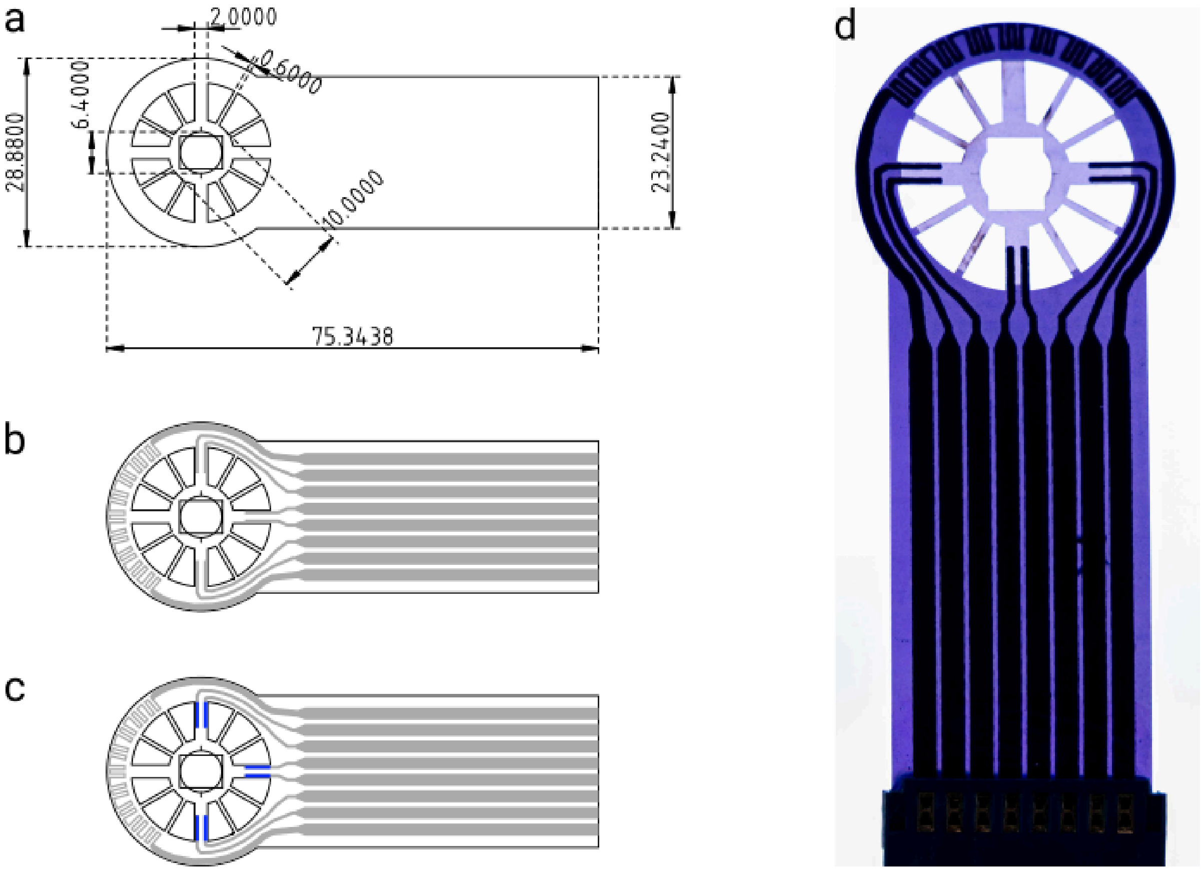

Figure S1: Geometry and printed layers of the printed impedance sensors. **(a)**: Shape and Dimensions used for final cutting using a laser as described in Section S2.1.1. **(b)**: Layout of the silver layer. The sensing electrodes protrude onto the wide spokes of the sensor, while the reference resistors' meandering traces loop around the left side of the sensor. **(c)**: Layout of the PEDOT:PSS layer. The sensing electrodes are fully covered by PEDOT:PSS to provide a high electrode capacitance, resulting in a good electrode-tissue coupling. **(d)**: Optical photograph of a finished sensor. By backlighting the sensor, the electrode structure is nicely visible behind the encapsulation layer.

S2.3 Experimental Procedure

S2.3.1 Sedation and Anesthesia

Animals are premedicated with azaperone (8 mg/kg IM; StresnilTM, Elanco, Germany), ketamine (20 mg/kg IM; 10% Ketamin, Medistar, Germany), and midazolam (0.2 mg/kg IM; Dormicum®, Roche, Germany). General anesthesia is induced using fentanyl (200 µg IV; fentadon®, Eurovet, Netherlands), propofol (100 mg IV; 2% Propofol MCT, Fresenius, Germany), and pancuronium (4 mg IV; Inresa, Germany), followed by endotracheal intubation (7 mm ID tube) and total intravenous anesthesia (TIVA) with continuous infusion of fentanyl (5–10 µg/kgBW/h), propofol (4–8 mg/kgBW/h), midazolam (0.1 mg/kgBW/h), and pancuronium (4–6 mg/h). Animals are placed supine on the operating table with all extremities restrained. Volume-controlled ventilation mode (Dr¨ager Primus®, Dr¨ager, Germany) is applied, with continuous monitoring of oxygen saturation, heart rate, respiratory minute volume, and end-expiratory CO2. A warming system is used throughout the procedure to prevent hypothermia. At the end of the experiment, animals are euthanized under continued anesthesia with an intravenous administration of pentobarbital (45 mg/kg; Release®, WDT, Germany)

S2.3.2 Surgical Model

After prepping the skin sterily, a midline laparotomy is performed. The ileocecal valve is identified, and side-to-end stapled anastomoses are recreated proximal to the valve. The mesentery is divided in an avascular plane, and the bowel is transected using monopolar cautery (ICC 300, Erbe Elektromedizin, Tubingen, Germany). The anvil of the circular stapler is inserted into the proximal end of the bowel (see Figure 1B.3) and secured with a pre-positioned purse-string suture (Vicryl 3.0, Ethicon®, Norderstedt, Germany). Through a small enterotomy on the distal bowel limb, the shaft of the stapler is inserted, and the sensor membrane is placed between the shaft and the anvil (see Figure 1B.4). The anastomosis is completed after advancing the shaft to meet the anvil and firing the stapler (see Figures 1B.4 and 5). Notably is the cutout of the innermost part of the membrane by the circular blade of the stapler, therefore providing a mucosal overlap intraluminally(see Figure 1B.2). The enterotomy is closed using a running suture (PDS 4.0, Ethicon®, Norderstedt, Germany). All sensor membranes are inspected for dislocation, and blood perfusion at the anastomotic site is verified using hyperspectral imaging (TIVITA®, Diaspective Vision, Pepelow, Germany). The intestine is then carefully repositioned into the abdominal cavity, and the operative field is temporarily covered with warm, moistened swabs. Baseline perfusion measurements of the intact anastomosis are recorded for at least 120 min. Subsequently, ischemia is induced in the anastomotic segment. For this purpose, vessel loops (ligatures) are pre-positioned around the supplying mesenteric vessels before starting the measurement. To induce ischemia, these ligatures are tied. Ischemia is verified and quantified using hyperspectral imaging. Following the onset of ischemia, measurements are continued for at least an additional 120 min to monitor the temporal progression of perfusion changes. Throughout this period, the abdomen stays covered with surgical swabs to preserve physiological conditions.

S2.3.3 Impedance Measurements

Bioimpedance is measured using a MCC USB-1208HS-4AO high-speed USB data acquisition (DAQ) device. A sinusoidal voltage signal with an amplitude of 0.1V is applied via each of the device's four analog outputs. This signal is routed through a fixed 2.2 kΩ reference resistor, followed by a sensor electrode pair or the substrate reference resistor. The resulting voltage drop across the tissue-interfacing electrodes or the reference resistor is recorded via four of the DAQ device's analog input channels. To capture a frequency-dependent impedance spectrum, measurements are conducted at logarithmically spaced frequencies ranging from 1 Hz to 25 kHz. Each full-spectrum measurement takes 30 s, and is followed immediately by the next, enabling continuous, real-time monitoring. The recorded time-domain signals are processed using a Fast Fourier Transformation (FFT) to extract the signal amplitude and phase shift for each frequency. Based on the known excitation voltage and the 2.2 kΩ reference resistor, the complex impedance of the tissue segment between the electrode pair is calculated.

To validate the accuracy of our custom DAQ-based impedance measurement system, a laboratory measurement is performed. For various dilutions between 1‰ and 1000‰ of Minimal essential Medium as a proxy for electrolytic body environments, impedance spectra are recorded using the DAQ-device setup (Figure S2a) and with a high-precision laboratory-grade potentiostat (PG-STAT302N, Metrohm) (Figure S2b) while using Silver/PEDOT:PSS electrodes. Measurements revealed similar impedance spectra for both, despite a slight mismatch in the phase information for low frequencies of the DAQ-device setup (see Figure S2).

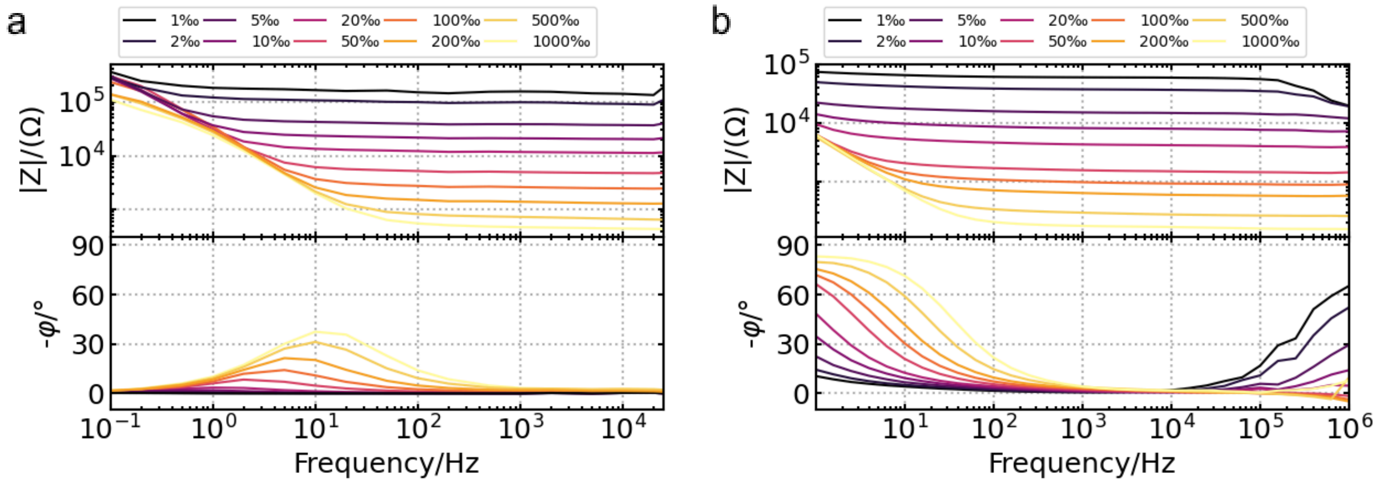

Figure S2: Comparison of recorded impedance spectra for Ag:PEDOT electrodes on various dilutions of Minimal Essential Medium (MEM, in permille) as a proxy for electrolytic body environments **(a)** recorded with our custom DAQ device Impedance measurement setup, 0.1V excitation amplitude vs **(b)** the same spectra recorded using the Metrohm PG-STAT302N (0.01V excitation amplitude). The different frequency ranges should be noted here: As described above, the frequency range of the DAQ devices is limited to around 25 kHz for 4-channel operation. For the Metrohm PG-STAT302N, frequencies < 1 Hz are prone to error and are not recorded in the spectra. For the impedance modulus (upper plots), in the overlapping frequency range, a similar behaviour is observed, with comparable absolute values. However, for the DAQ devices, a flattening of the curve is observed for low frequencies, indicating the limited dynamic range of the single resistance measurement setup. For the recorded phase information, some differences are observable, mainly for frequencies < 100 Hz.

S3 Detailed Data Discussion of all Anastomosis Sensors

As explained in the Main document, we experienced quite a large variation in our measured data. Parts of this variance are due to slight differences and deviations from our presented experimental protocol from experiments prior to the last two experiments (see ethical statement in the main document).
In this section, we will try to give some insight into these changes, how they influence the data, and also give and explain the manually assigned “Data confidence ratings” for each sensor of each anastomosis.

S3.1 Animal 5, Day of Operation: 29.1.2025

First, we start with the anastomoses 1-3, for which measurements were performed at one day simultaneously in one animal. The data is presented in Figures S2 to S4.
Compared to the measurement protocol presented in the main document, there are four main differences.

1. For the induction of ischemia, surgical clamps were used to clamp off the supplying mesenteric vessels. Consequently, this procedure requires a lot of mechanical manipulation of the intestine, resulting in changes in the electrode-tissue contact, which in turn leads to sudden drops or increases in the impedance data.
2. Hyperspectral imaging was performed immediately before ischemia induction, and at the end of 2 hours, while the impedance measurement was still running. This added to the required mechanical manipulations, leading to the problems described above.
   For the following experiments, hyperspectral imaging was performed before starting and after ending the impedance measurements.
3. Both before and after ischemia induction, a 40 °C hot electrolyte solution was poured onto the abdominal swabs roughly every 30 minutes. This causes visible drops in impedance across all recorded frequencies, which makes it difficult to separate actual signals from unintended interference.
4. Sensors were soaked overnight in PBS solution for ~10 h before implantation. As the sensing fingers are not protected by an additional polydioxanone layer, adhesion of the sensors decreases. The mechanical stress caused by stapling can potentially lead to the detachment of the whole sensing setup. However, small “stubs” of the sensor, which are covered by the PDO, may remain intact, leading to a reduced sensor capacity and subsequent changes to the recorded impedance spectra. For the later experiments, this soaking was omitted.

In addition to these differences, we observed incomplete ischemia at anastomoses 1 and 2. We decided to improve the blockage of the mesenteric vessels while continuing the recording of impedance spectra.
Despite these changes and difficulties, we try to extract valuable information from the recorded data to make the most use of the sacrificed animals under the 3Rs principles. In the following figures, the data, problems, extracted information for pooled analysis, and confidence ratings will be explained. All figures follow the same structure, with all raw data and heatmaps in one plot for each measuring channel. Channel 0 (CH 0) is on the top row, left, and represents the reference resistor in all cases. Channels A, B, and C correspond to the individual sensor pairs of each anastomosis sensor, with CH A top right, CH B bottom left, and CH C bottom right.

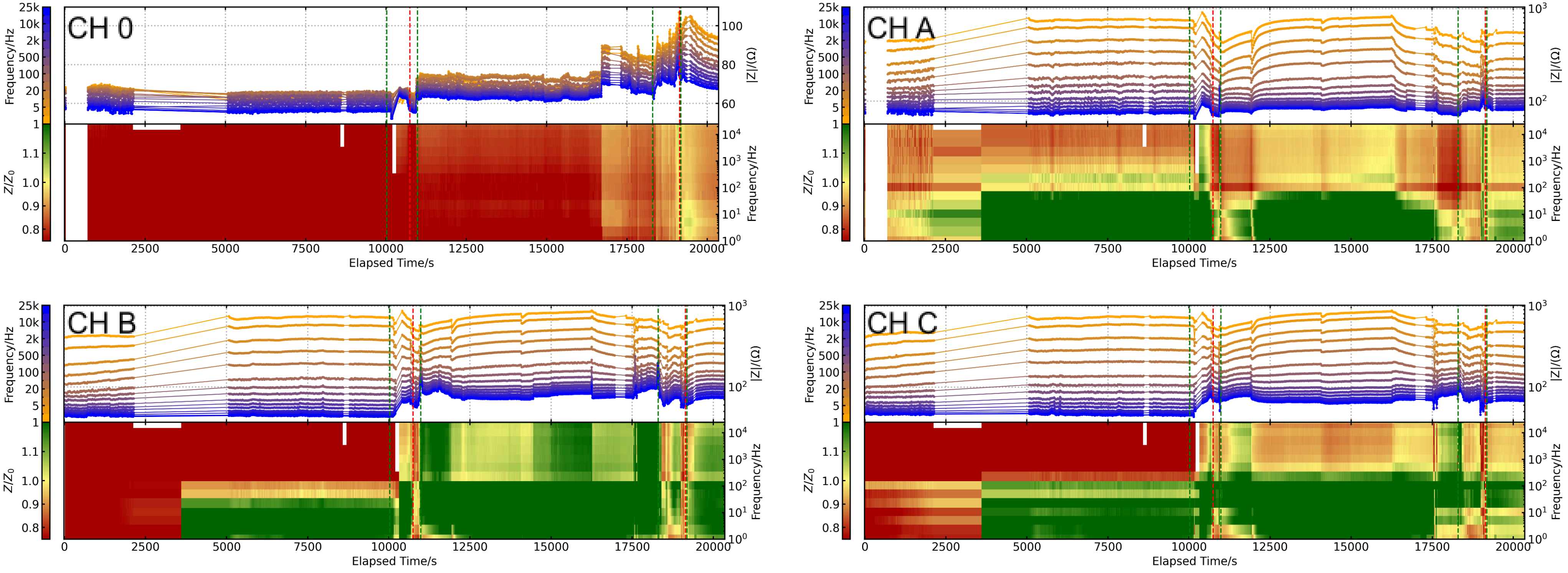

Figure S3: Complete Dataset of Anastomosis 1: The first 700 seconds are skipped for CH 0 and CH C, due to contact problems. Unfortunately, we experienced an at first unnoticed bug in the measuring script, which caused a long gap in the impedance data between 2500-5000 s.
Until the first ischemia induction, all baselines show stable resistances, with a noise level of 1-2% depending on the frequency between 5000 and 10000 seconds. During the manipulation period - marked by the green vertical dashed lines - shifts in impedance are observed, making the baseline obsolete and impossible to compare to. However, if one assumes the impedance value at the end of the manipulation period to be the reference value, changes in impedance, both drops and increases, are observable in the first 10 minutes after ischemia induction, but are also inconsistent. One effect nicely visible here is the drops in impedance due to the pouring of electrolyte solution onto the abdominal swabs around the 12000 s and 14000 s mark. Unfortunately, we are unable to place high trust in the data in this timeframe. However, as explained before, a second effort was made to improve the blood supply blockage at around 19000 s. For all channels, a subsequent change in impedance is observed. For CH B and C, a gradual rise is observed in all frequencies. For CH A, however, impedance increases for low frequencies, while a small drop is observed for high frequencies. We take this as an indication that this change is more significant than the observed changes in B and C. We rate CH A at 75% confidence, while CHs B and C receive only a rating of 60%

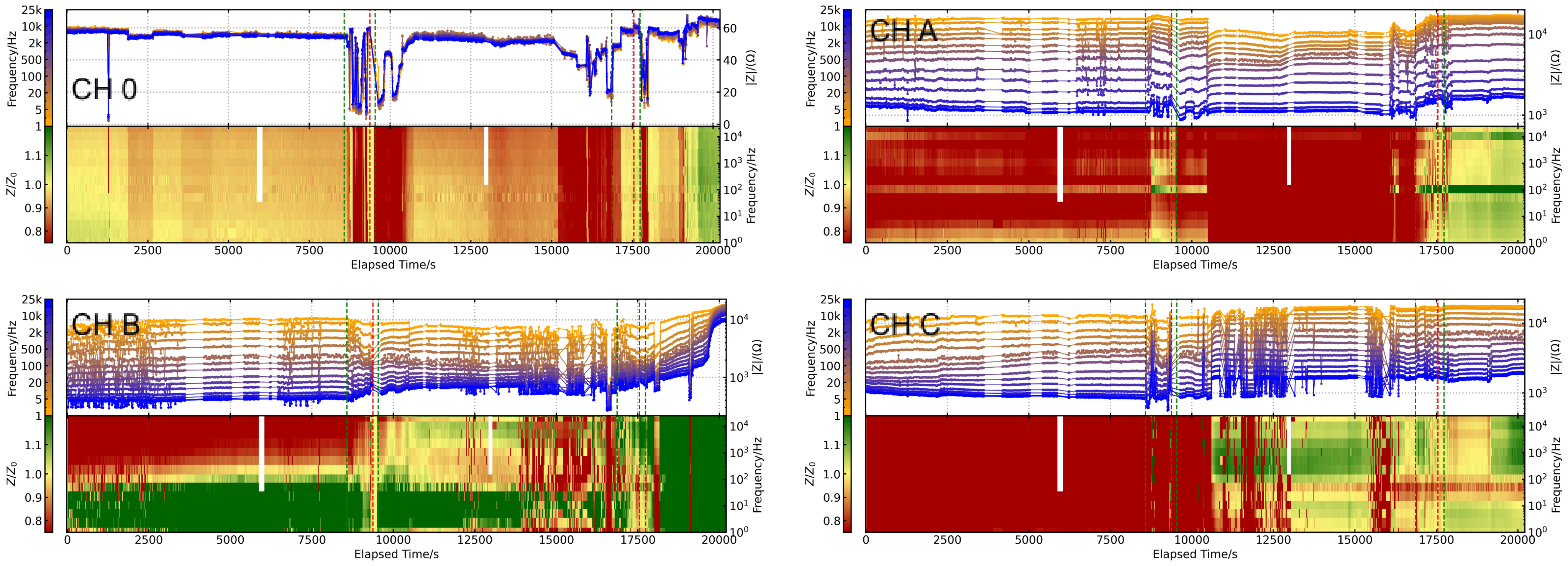

Figure S4: Complete Dataset of Anastomosis 2: The reference resistor at CH 0 shows a lot of jumps and changes, indicating some issues with this multisensor set. All mechanical manipulations are visible there, as sudden changes in the observed resistance. Unfortunately, all sensor data is quite noisy, especially after ischemia induction. Before that, in the baseline establishment period between 0 and 7000 s, noise levels are comparably high with ~5% for CH A, and ~10% for CH B and C. During mechanical manipulation for ischemia induction, noise is high, and impedance levels shift, similar to Anastomosis 1. After the end of the manipulation work, the impedance levels rise and stabilize for low frequencies around 10000 s. At the same time, a distinct, quick rise, followed by a rapid decrease in impedance, is observed. Similarly to before, the low frequencies show a different behaviour in this time frame, so we give a confidence rating to all three sensor channels, CH A, B, and C, of 75%

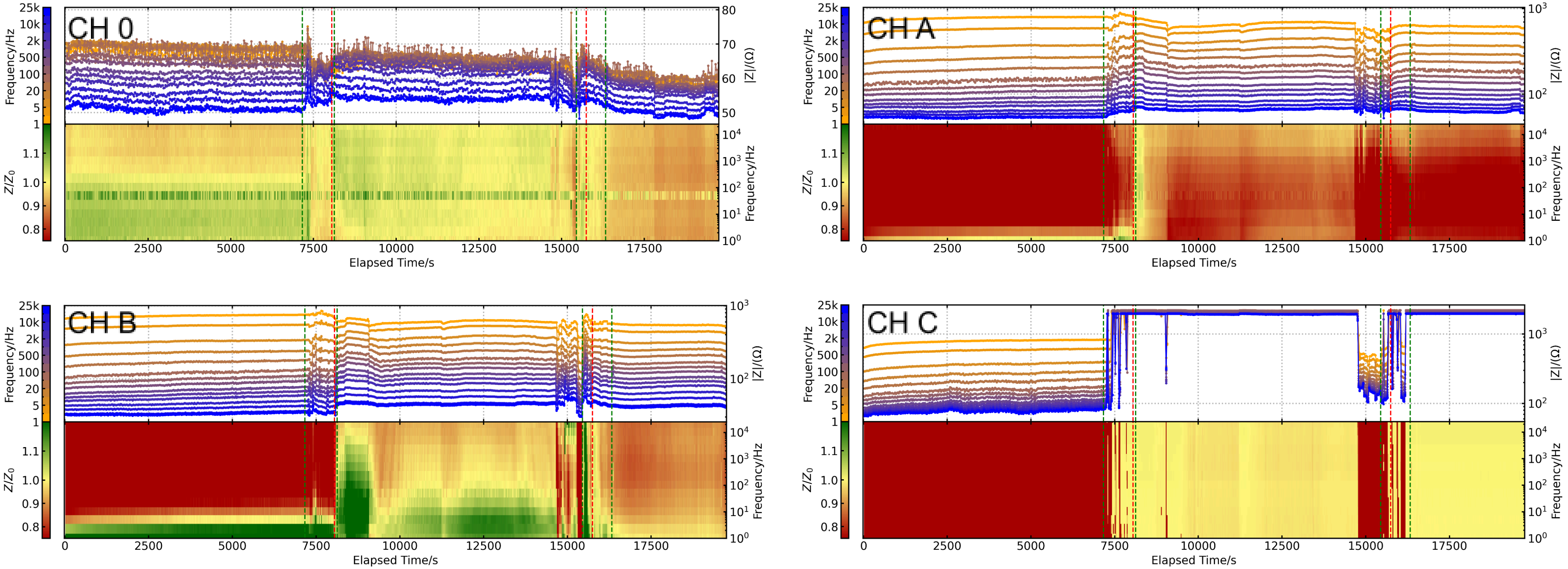

Figure S5: Complete Dataset of Anastomosis 3: The reference resistor CH 0 keeps a relatively stable baseline impedance, with just small changes in phases of strong mechanical manipulation of the baseline. These manipulations are also visible in the data for CH A and B. For CH C, during the first manipulation, some kind of “short circuit” happened, which is seen in the loss of frequency dependence of the impedance at 7500 s. Inspection at the end of the experiment revealed broken sensors for this channel. This data, unfortunately, is unusable, resulting in a trust rating of 0% for CH C. For CH A and B, the baseline and, in general, the data are quite stable. Noise levels of the baseline between 0 s and 7000 s lie around 1-6% depending on the frequency. For CH A, we observe an impedance drop in the high frequencies around 400 s after the first ischemia induction at 8000 s. While the impedance is slowly decreasing for all frequencies in this time range, the sudden impedance drop is only observed in high frequencies. From this, we assign CH A a confidence rating of 75%, as the baseline shifted significantly during mechanical manipulation. For CH B, also sudden impedance changes are visible in the data, but here the changes occur at all frequencies, which indicates a different reason for the change than the ischemia induction. Also, at the second clamping, no significance can be seen. We assign this data of CH B a confidence rating of 50%.

S3.2 Animal 6, Day of Operation: 30.1.2025

For this animal, we added a fourth anastomosis, which was used to validate the DAQ Device measuring setup using the Metrohm Autolab PG-STAT302N. We measure anastomosis 7 CH C using this device.
In the following figures, we present the data of anastomoses 4-7. This experiment session followed the protocol presented in the main document, with ligature loops blocking the mesenteric vessels. Hyperspectral imaging was done before the start and after the end of the impedance measurements to minimize mechanical manipulation and influences on the recorded data. Sensors were not soaked in PBS overnight, which results in an initial decrease of impedance in a certain “acclimatization period” during the first ~30 minutes. This effect was described in [1], where printed silver lines on PDO have an initial decrease in resistance after immersion in an aqueous and electrolytic environment. Additionally, no electrolyte solution was poured onto abdominal swabs during surgery to eliminate artifacts in the recorded data. In total, measurements in this animal are more stable and more reliable than any data before, yet not all data is usable and 100% reliable.
One important thing to note is the use of anastomosis 5 as a reference measurement for around 40 minutes during the post-ischemia period of anastomoses 4, 6, and 7. For anastomosis 5, the prepared vessel ligatures were not tied during the work period for 4,6, and 7, but later in a quick working period around 10000 s.

[1] Jaekel u. a., „Materials and Fabrication Methods for Fully Resorbable Implantable Sensors on Medically Certified Polydioxanone“.

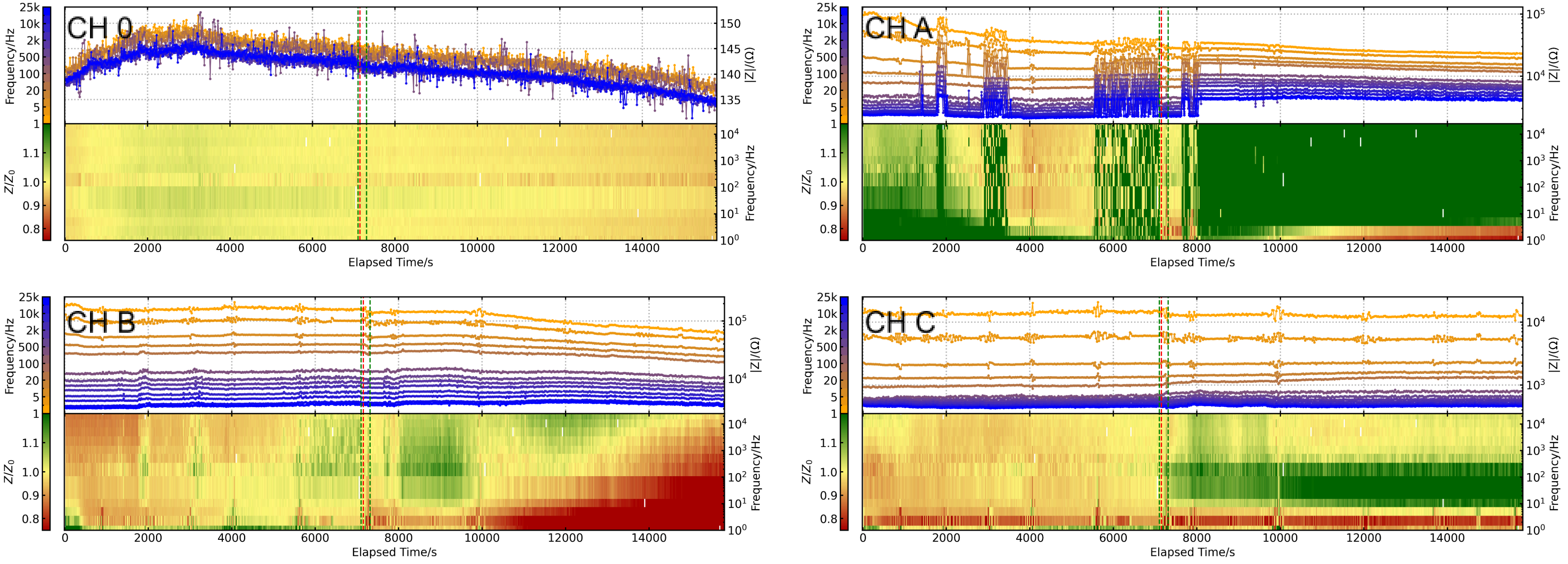

Figure S6: Complete Dataset of Anastomosis 4: CH 0 shows a good, stable reference resistance, which decreases during the scope of experimentation. CH A unfortunately shows strong noise and unstable baselines, indicating some problems with this sensor. Additionally, the measured impedance lies around 2 orders of magnitude above the other measurements (10^2-10^3 for measurements in other anastomoses, while 10^4-10^5 for this sensor). Most likely, the contact of the sensor is broken at some point, though it was not visible to the naked eye. CH B exhibits the same order of magnitude as CH A, unlike the sensors of other anastomoses. Potentially, we can see a small drop in impedance around 8000 s, followed by an increase. However, as the order of magnitude does not match the other anastomoses, we can not assign a high confidence rating here, and give it a rating of 60%. For CH C, baselines are quite stable, the impedance order of magnitude fits well. Here, however, signal strength is low. No drop in impedance is observed, but instead impedance rises for high frequencies at 8000 s and drops again at the 10000 s mark, contrary to the signal presented in the main document Figure 2. Towards the end of the measurement period, a gradual increase can be observed. We assign this channel a confidence rating of 80%, being more trustworthy than the data of anastomoses 1-3, but as the other channels show a strange behaviour, we can not assign this sensor a good rating in general.

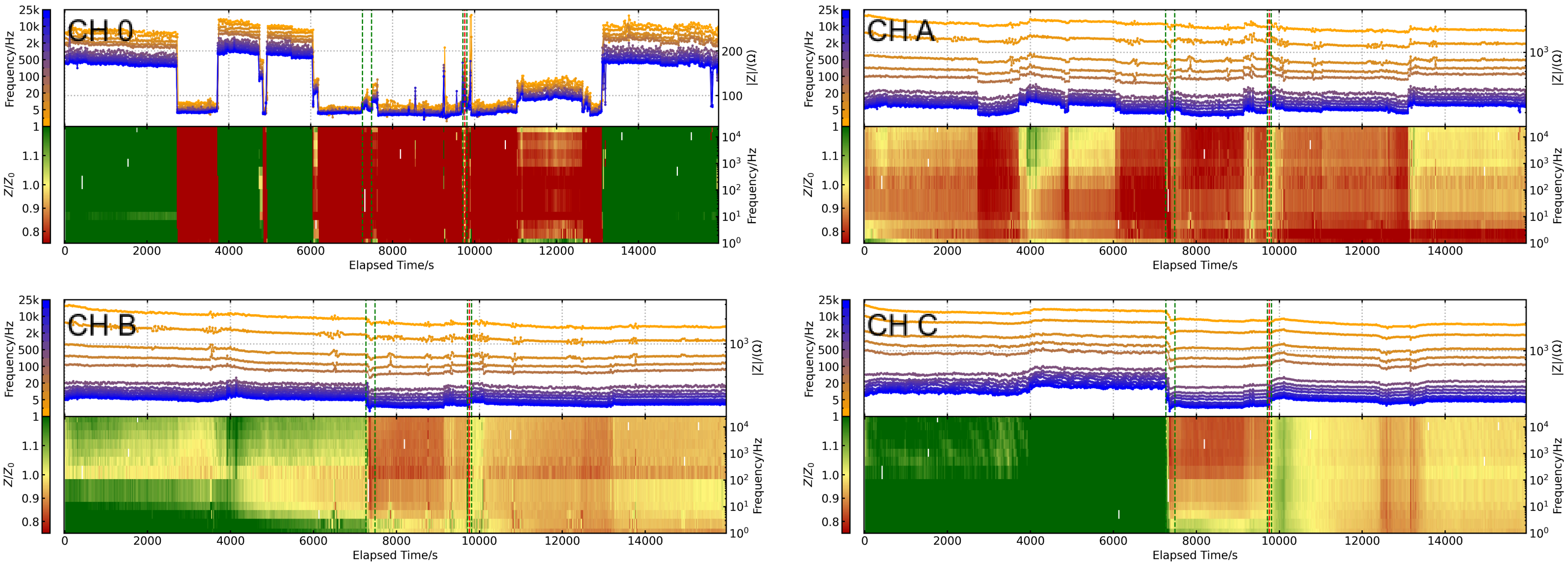

Figure S7: Complete Dataset of Anastomosis 5: One trend visible for the whole dataset here is a lot of sudden jumps in impedance for all channels. Here, the reference resistor CH 0 and sensor CH A exhibit this trend the strongest, with most jumps occurring in both channels. We are unsure why this is, but this renders the data less trustworthy. While an impedance drop is visible in the data for CH A, we can only assign a confidence level of 50% for this channel due to the large number of jumps in impedance. CH B and CH C have a more stable baseline, and the jumps are less sudden, but still, the baseline changes significantly during the pre-ischemic period. Regarding the signals after ischemia, CH B exhibits a small, but noticeable drop 400 s after vessel closure. A similar behaviour is seen in CH C. However, here the impedance drop occurs for all frequencies simultaneously. Both CH receive a confidence rating of 75%. Regarding the use of this anastomosis as a reference sensor, and disregarding CH A, impedance levels remain constant after ischemia induction in the other anastomoses, proving the substantiality of the recorded signals for anastomoses 4,6, and 7.

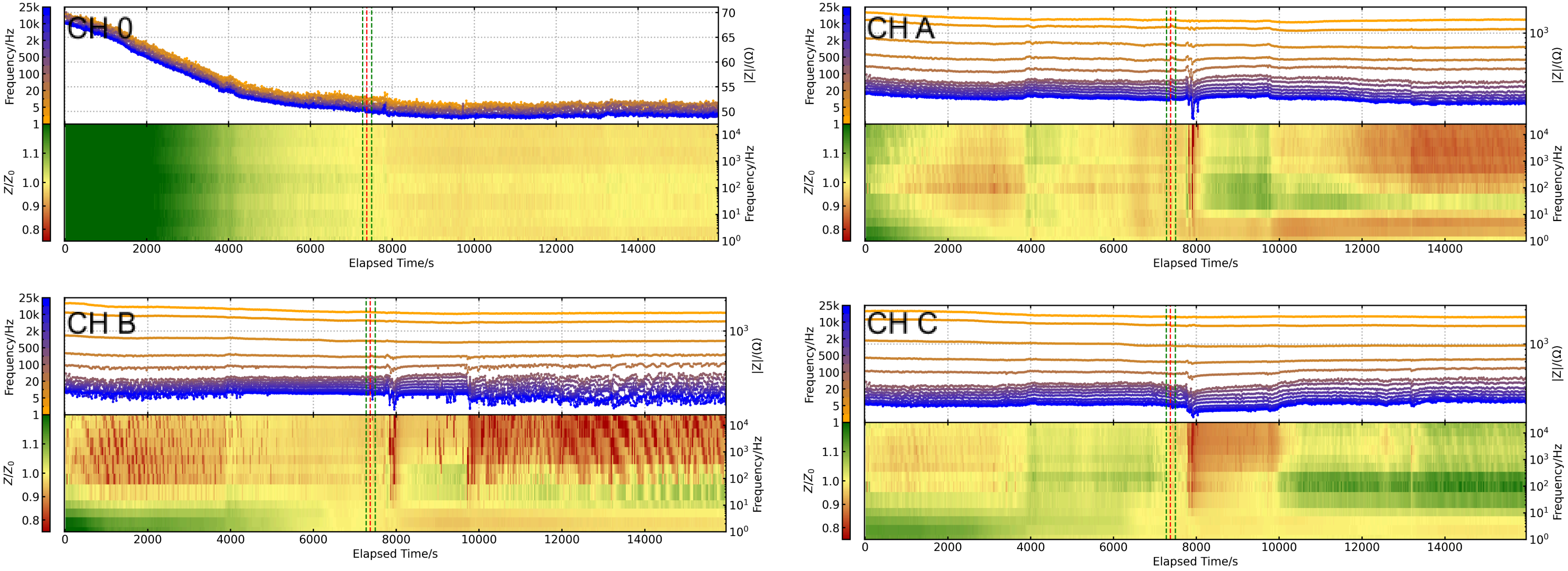

Figure S8: Complete Dataset of Anastomosis 6: This is the anastomosis presented in the main document. CH 0 exhibits a pronounced acclimatization period in accordance with[2] as described before. In all channels, CH A, B, and C, a pronounced drop is observed in high frequencies roughly 600 s after ischemia induction. The behavior after this drop differs slightly. For CH A, impedance increases to slightly higher levels than before ischemia, but slowly decreases until the end of the measuring period. For CH B, a recovery of impedance is observed. However, noise levels increase drastically after 10000 s, which makes the interpretation of the data in this timeframe difficult. For CH C, impedance stays at a lower level than before the ischemia, but recovers after ~30 minutes. Afterwards, impedance levels gradually increase. All data is assigned a confidence rating of 100%.

[2] Jaekel u. a., „Materials and Fabrication Methods for Fully Resorbable Implantable Sensors on Medically Certified Polydioxanone".

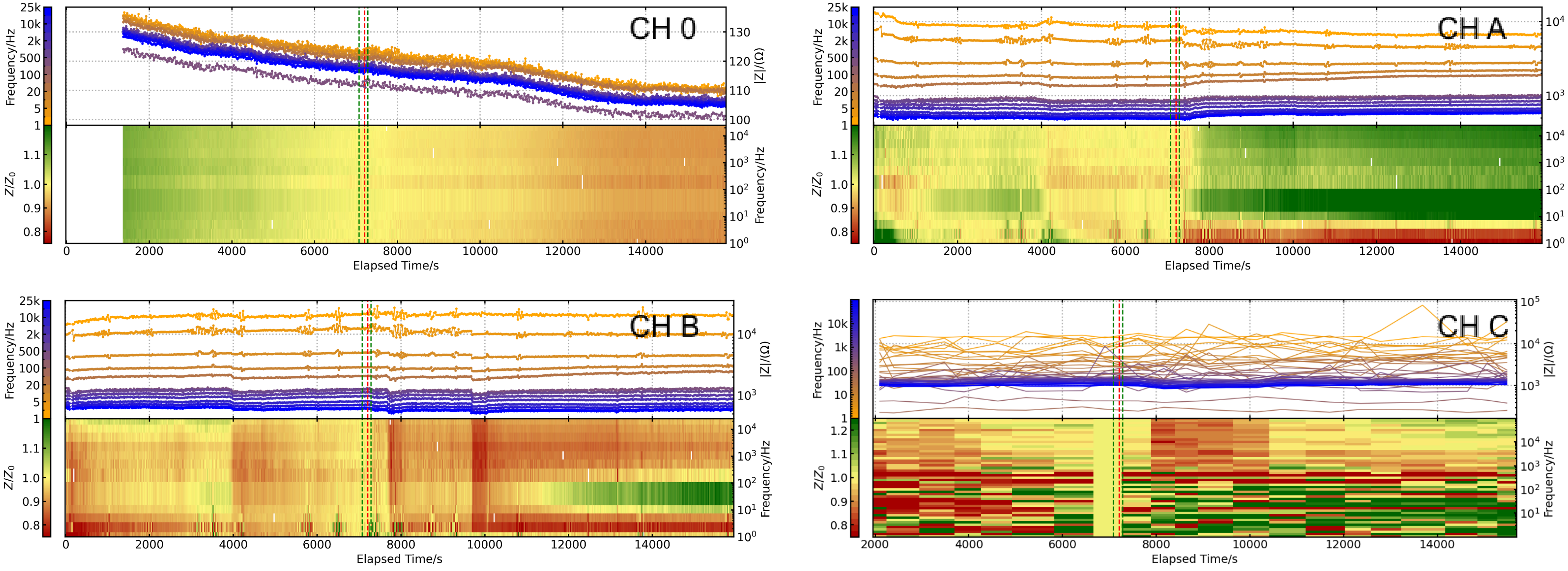

Figure S9: Complete Dataset of Anastomosis 7: The reference resistor CH 0 shows a similar acclimatization behaviour as seen previously in Figure S7. Data in the beginning was skipped due to a missing electrical contact at the DAQ devices. CH A shows a stable baseline with ~1% noise. After ischemia induction, impedance levels rise for high frequencies, while they drop for low frequencies. Despite the unusual behavior, we set a high confidence rating of 100% for this channel. For CH B, the baseline is slightly less stable with ~2-4% noise. Still, at the same time as for anastomosis 6 around 8000 s, 600 s after ischemia induction, the impedance drops for high frequencies, while remaining relatively stable for low frequencies. Impedance levels recover slightly, but do not rise to pre-ischemic levels. Impedances drop again at around 10000 s, after which a slight rise is observed again, similar to Figure S7, CH C, but without rising above pre-ischemic levels. This sensor is rated with a confidence rating of 100% as well. CH C shows the high-precision validation measurement data recorded with the Metrohm PG-STAT302N. This data was discussed in detail in the main document. Also, here, the confidence rating is set as 100%.
One interesting thing to note here is the periodically occurring noise for low frequencies observed in CH A and CH B. The times when noise increases coincide with the starting times of the PG-STAT302N measurements. The low frequencies pick up the signals from the device, which in turn leads to noise in the recorded impedance. This noise is partially picked up by the sensors of other anastomoses.

This concludes all usable data. Even earlier measurements are not worth showing, as data quality is limited. Due to the complexity of the experiment, we adjusted the protocol, addressing our technical and surgical problems. This long process, accompanied by technological development, ultimately led to the measurements presented in this paper. Despite all problems, we are confident in the measurement protocol and the data presented in this manuscript, and believe that further experiments will only validate this data further.

S4 Lifetime of electrodes

The lifetime of electrodes in 37°C minimal essential medium (MEM) was determined. Printed resistors, both encapsulated and unencapsulated, were immersed in ~100ml of MEM, and resistance was monitored once approximately every 10 minutes. Resistors are considered broken once the resistance increases to a factor of 10 of the original Resistance R_0. Unencapsulated resistors break down on average after 11.6 days, while the encapsulated resistors remain functional even after 20 days of Immersion. The measurement was stopped, as this lifetime is already sufficient for our application, where a functional lifetime of 10 days is required.

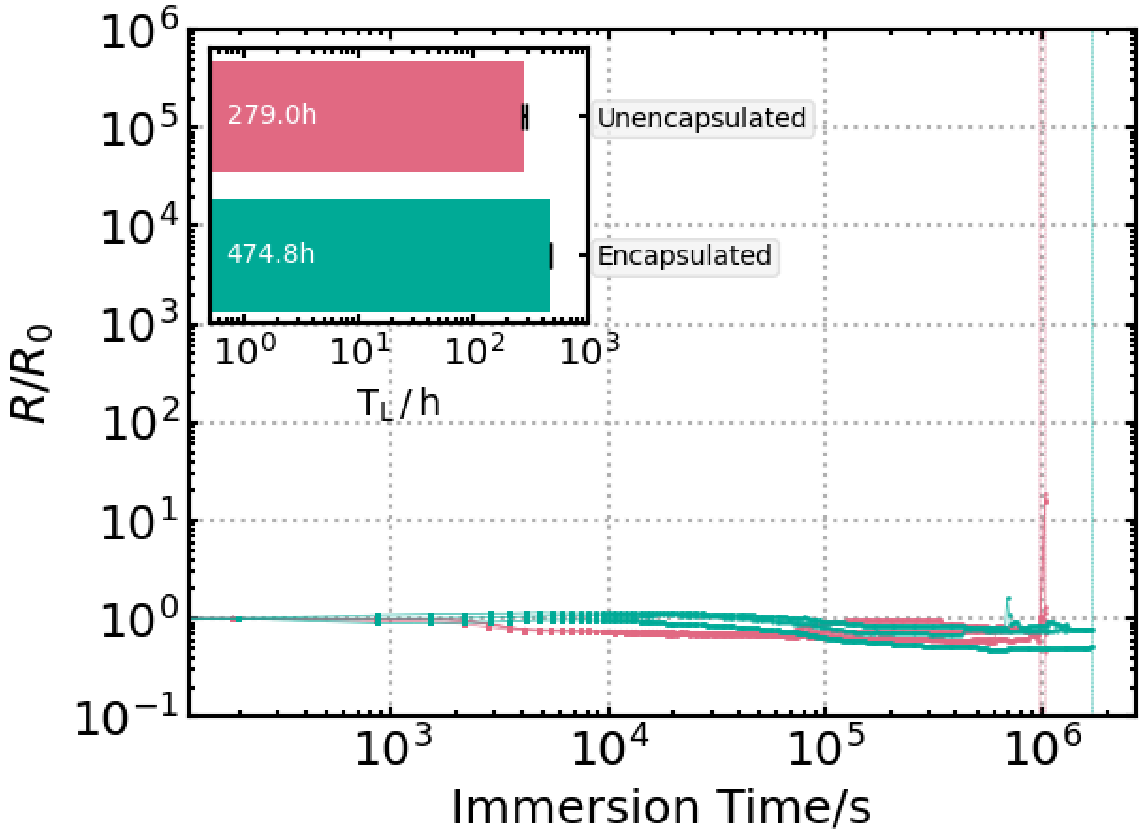

Figure S10: Lifetime of encapsulated (green) and unencapsulated (red) printed silver resistors immersed in 37°C minimal essential medium (MEM) as a proxy for complex electrolytic environments found in the body. Unencapsulated resistors break down after approximately 279 hours (11.6 days), while encapsulated resistors remain functional after 470 hours (20 days), after which the experiment was stopped.

S5 Feasibility of membrane integration

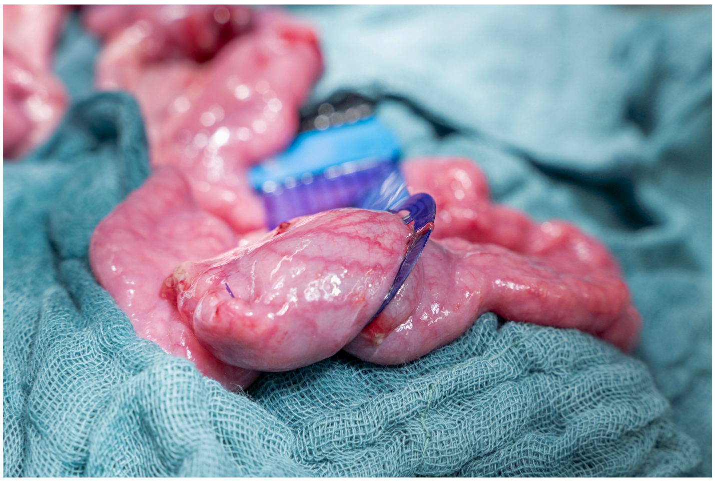

Figure S11: We present another angle in a high-resolution image of an implanted sensor in an ileal end-to-side anastomosis immediately after implantation. The sensor spokes are well integrated into the anastomosis, while the outer ring remains externally positioned.

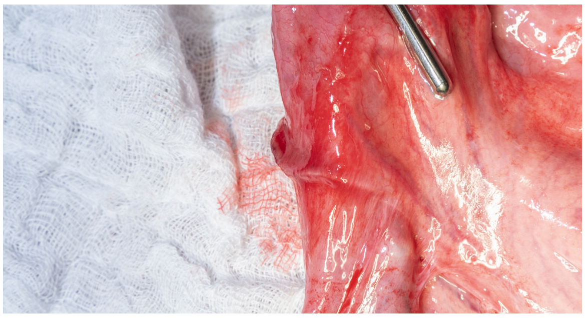

Figure S12: This image shows the incorporated bioresorbable membrane on the seventh postoperative day. A visual remnant is still identifiable in its orthotopic position corresponding to the implantation site. The anastomosis appears intact, and the mesentery, including the supplying blood vessels, is seamlessly aligned.

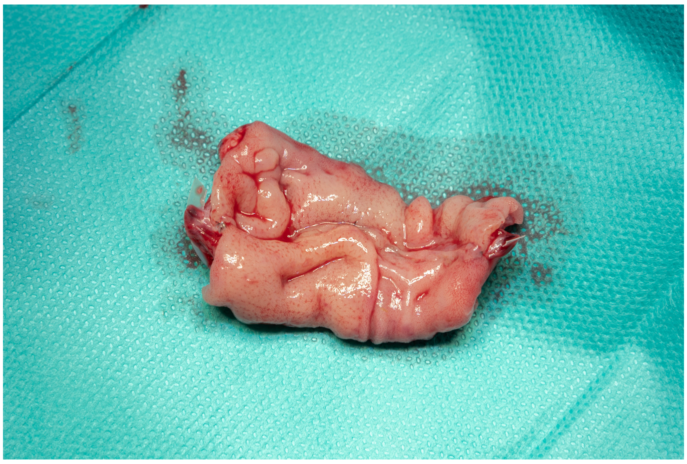

Figure S13: This image showcases the resected and longitudinally opened anastomosis following sensor implantation, demonstrating complete mucosal closure. Although the sensor spokes penetrate the intestinal wall, the mucosal surface overlaps the innermost components, thereby minimizing any potential adverse effects on anastomotic healing.
